# Negr1 Deficiency Modulates Sex-Specific Neurobehavioral Adaptations to Social Isolation

**DOI:** 10.3390/brainsci15121286

**Published:** 2025-11-29

**Authors:** Arpana Reinsberg, Katyayani Singh, Mohan Jayaram, Kaie Mikheim, Mari-Anne Philips, Eero Vasar

**Affiliations:** Institute of Biomedicine and Translational Medicine, Department of Physiology, University of Tartu, 19 Ravila Street, 50411 Tartu, Estonia

**Keywords:** IgLONs, Negr1, stress, social isolation, behavior, gene expression

## Abstract

**Objective**: Neuronal growth regulator 1 (*Negr1*) is a GPI-anchored neuronal cell adhesion molecule of the IgLON superfamily associated with multiple psychiatric disorders. This study aimed to investigate behavioral and molecular adaptations to social isolation (SI) stress in *Negr1*-deficient (*Negr1^−/−^*) mice. **Methods**: Male and female *Negr1^−/−^* and wild-type (Wt) mice (n = 10 per group) were exposed to two weeks of SI or group housing (Ctl). Behavioral assays assessed exploratory and anxiety-like behavior. Gene expression analyses in the prefrontal cortex and hippocampus were performed using RT-qPCR, focusing on GABAergic, neurotrophic, and IgLON family genes. **Results**: SI-induced weight loss in *Negr1^−/−^* mice compared to Wt was evident in both sexes but more pronounced in males. Behaviorally, SI Wt males showed stress-induced hyperactivity compared to Ctl Wt, whereas SI *Negr1^−/−^* males exhibited blunted exploratory behavior relative to SI Wt in the open field test (OFT). *Negr1^−/−^* females showed reduced exploration in the elevated plus maze (EPM), suggesting increased anxiety. Hippocampal *Pvalb* was downregulated in SI *Negr1^−/−^* mice of both sexes compared to Wts, with a stronger decrease in males, indicating heightened male vulnerability in GABAergic interneuron function. In males, SI reduced hippocampal *Bdnf* in both genotypes, whereas *Ntrk2* (TrkB) upregulation occurred only in *Negr1^−/−^* mice, suggesting a genotype-specific compensatory response. Hippocampal expression of *Fgfr2* and IgLON members (*Ntm1a/1b*, *Lsamp1a/1b*) was increased in SI *Negr1^−/−^* males compared to SI Wt, with minimal changes in females. **Conclusions**: *Negr1* deficiency leads to sex-specific behavioral and molecular adaptations to social isolation stress, highlighting the role of *Negr1* in modulating neurotrophic and GABAergic signaling pathways under adverse environmental conditions.

## 1. Introduction

Neuronal growth regulator 1 (Negr1) is a member of the IgLON family within the immunoglobulin superfamily of cell adhesion molecules. The IgLON family comprises limbic system-associated membrane protein (Lsamp), opioid-binding protein/cell adhesion molecule-like (Opcml), neurotrimin (Ntm), neuronal growth regulator 1 (Negr1), and IgLON5. These are glycosylphosphatidylinositol (GPI)-anchored membrane proteins that mediate cell–cell interactions essential for neural development and synaptic organization [1,2,3]. IgLONs are highly and specifically expressed throughout the central nervous system during both development and adulthood [4]. At the cellular level, Negr1 forms both cis-interactions (with itself or other IgLON family members on the same cell membrane) and trans-interactions (across the synaptic cleft with binding partners on opposing membranes) [5]. Recently, IgLONs have been identified as extracellular interaction partners of AMPA-type glutamate receptors (AMPARs), with associations that are dynamically regulated during synaptic plasticity [6].

Members of the IgLON family have been implicated in key neurodevelopmental processes across the central and peripheral nervous systems, including neuritogenesis, axon guidance, synaptogenesis, dendritic arborization, and myelination across multiple brain regions [1,2,3,7,8]. More recent studies using transgenic mouse models have highlighted the role of Negr1 in adult brain neurogenesis (hippocampus and olfactory) and its association with affective behaviors and neuropsychiatric disorders such as anxiety and depression [9,10,11]. Consequently, there is an increasing interest in delineating the molecular and cellular mechanisms through which Negr1 influences neuronal connectivity, circuit maturation, and behavioral regulation.

Genome-wide association studies (GWAS) have repeatedly identified NEGR1 as a gene of interest in major depressive disorder, schizophrenia, and body mass index across diverse populations [12,13,14,15]. Variants in NEGR1 have also been associated with alterations in white matter integrity in individuals with psychiatric disorders, independent of obesity-related traits, and with susceptibility to eating disorders [16,17]. Several studies have reported a modest but consistent reduction in body mass in mice lacking functional Negr1 when maintained on a standard diet [18,19,20]. However, increased weight gain compared to wild-type mice has been observed in *Negr1^−/−^* male mice under a high-fat diet [19], suggesting altered metabolic homeostasis in these animals.

Histological and imaging analyses of *Negr1^−/−^* mouse brain have revealed anatomical abnormalities such as widespread volumetric reductions in cortical and subcortical structures, including the hippocampus [21,22,23]. These structural changes are accompanied by impaired dendritic arborization, altered expression of genes involved in synaptogenesis and plasticity, and behavioral abnormalities, such as hyperactivity, reduced social interaction, and increased anxiety- and depression-like behaviors. The hippocampus appears to be particularly affected, exhibiting reduced volume, loss of parvalbumin (*Pvalb*)-expressing interneurons, and disrupted synaptic organization [21]. Pvalb interneurons, a subset of fast-spiking GABAergic neurons, play a pivotal role in maintaining the excitation–inhibition (E/I) balance in cortical and hippocampal circuits. They are characterized by their expression of *Pvalb*, a calcium-binding protein that modulates intracellular calcium buffering and kinetics, enabling rapid and precisely timed inhibitory signaling [24,25].

Loss or dysfunction of Pvalb interneurons compromises GABAergic inhibition, leading to impaired gamma oscillations and disrupted synchronization of pyramidal neuron networks. Such disturbances in E/I homeostasis are strongly linked to cognitive deficits, emotional dysregulation, and increased vulnerability to psychiatric disorders, including schizophrenia and major depression [26,27]. In *Negr1^−/−^* mice, the reduction in Pvalb interneurons within the Cornu Ammonis (CA) and Dentate Gyrus (DG) regions likely contributes to hippocampal hyperexcitability and altered stress reactivity. *Negr1^−/−^* mice have already displayed heightened sensitivity to amphetamine, suggesting altered dopaminergic regulation and enhanced responsiveness to stimulatory challenges [28]. These findings indicate that *Negr1* plays an important role in modulating behavioral adaptation.

In vitro, Negr1—initially identified as Kilon—has been shown to regulate neuronal synapse formation in rat hippocampal cell lines [29] and impair neuronal maturation in murine cortical cell cultures, reinforcing its importance in synaptic development and circuit stabilization [30].

Negr1 contributes to the establishment of functional neural networks through its interactions with other neural adhesion and signaling molecules. Contactin-1 (Cntn1) has been repeatedly identified as a physical binding partner of Negr1 [25,31]. Beyond cell adhesion, emerging evidence suggests that Negr1 modulates neurotrophic and synaptogenic signaling cascades. For instance, Negr1 transcript levels are upregulated in *Tcf4*-deficient neurons [32] and in the hippocampus of *Tcf4^+/−^* mice [33], suggesting that *Tcf4* normally represses *Negr1* expression.

Moreover, Negr1 interacts with lipocalin-2 (Lcn2) and fibroblast growth factor receptor 2 (Fgfr2), forming complexes that regulate neuronal growth, differentiation, and synaptic remodeling [25,34] Recent findings also demonstrate that Negr1 modulates G protein–coupled receptor (GPCR)-mediated Ca^2+^ signaling and synaptogenesis in salivary glands, implying a broader role in regulating sympathetic nervous system activity and neurotransmitter release—mechanisms relevant to stress responses and altered salivation observed in depressive states [35].

Despite extensive research on stress-induced behavioral phenotypes, the molecular mechanisms linking Negr1 to stress susceptibility remain poorly understood. Standard paradigms used to model stress in rodents, including restraint stress, foot shock, predator odor exposure, maternal separation, witnessing stress, chronic unpredictable stress and social isolation [35,36,37,38,39], highlight the multifaceted impact of stress on neural circuits. However, the contribution of Negr1 to the transcriptional and cellular adaptations under such conditions has not been systematically explored.

Building upon current evidence implicating *Negr1* in neurodevelopment, synaptic plasticity, and behavior, this study aimed to investigate how social isolation affects psychoemotional function and neural alterations in *Negr1^−/−^* mice. Both male and female mice were included, addressing the prevailing male bias in previous research. We hypothesized that *Negr1^−/−^* mice would exhibit increased vulnerability to social isolation stress, as evidenced by altered gene expression and behavioral outcomes.

To experimentally assess the impact of *Negr1* deficiency on behavioral and molecular adaptations to social isolation stress, we employed an integrative behavioral–molecular approach in male and female wild-type (Wt) and *Negr1^−/−^* mice. By combining behavioral assays sensitive to anxiety and exploratory activity—such as the elevated plus maze, open field test, and home-cage monitoring in the PhenoTyper^®^ system—with targeted RT-qPCR analyses, we aimed to identify Negr1-dependent transcriptional responses within key limbic and cortical regions, particularly the prefrontal cortex and hippocampus. These experiments were designed to test whether the absence of Negr1, combined with social isolation, alters the expression of genes critical for GABAergic transmission (e.g., *Gad1*, *Gad2*, *Pvalb*), synaptic adhesion and signaling (*IgLONs*, *Cntn1*, *Fgfr2*), and neurotrophic regulation (*Bdnf*, *TrkB*, *Tcf4*). 

## 2. Materials and Methods

### 2.1. Animals and Ethics

Wild-type (Wt) and homozygous *Negr1*-deficient (*Negr1^−/−^*) mice littermates, derived from (129S5/SvEvBrd × C57BL/6N) × (129S5/SvEvBrd × C57BL/6N) background of the F2 generation were used for this study. The generation of this line has been previously characterized [18]. Mice were housed at the animal facility of the Institute of Biomedicine and Translational Medicine, University of Tartu, Estonia, and were maintained in cages measuring 42.5 cm (L) × 26.6 cm (W) × 15.5 cm (H), with 10 animals per cage, and provided with 2 cm of aspen bedding (Tapvei, Estonia) and 0.5 l of aspen nesting material (Tapvei, Estonia). Food (R70, Lactamin AB, Kimstad, Sweden). Water (in-house autoclaved) was provided ad libitum, with a 12:12 h light and dark cycle (lights off at 19:00), at a temperature of 22 ± 1 °C and relative humidity of 50–60%. For our experiment, estrous cycle stages were not monitored in female mice during behavioral testing or tissue collection. The use of mice was conducted in accordance with the regulations and guidelines approved by the Laboratory Animal Center at the Institute of Biomedicine and Translational Medicine, University of Tartu, Estonia. All animal procedures were conducted in accordance with the European Communities Directive (2010/63/EU) with permit (dated 17 April 2023, no. 1.2-17/168) from the Estonian National Board of Animal Experiments. We confirm that this study is reported in accordance with the ARRIVE (Animal Research: Reporting of In Vivo Experiments) guidelines as outlined at https://arriveguidelines.org (accessed on 26 October 2025).

### 2.2. Social Isolation

The social isolation (SI) cohort included 80 mice (40 males and 40 females) aged 4.5 ± 0.7 months. At the time of weaning, postnatal day 21 both control and SI mice were group-housed (10 per cage). In this study, as SI was our treatment and *Negr1^−/−^* our genotype, mice were divided into four groups based on genotype and treatment: control wild-type (Ctl Wt), control Negr1 knockout (Ctl *Negr1^−/−^*), socially isolated wild-type (SI Wt), and socially isolated Negr1 knockout (SI *Negr1^−/−^*). Control mice continued group-housing (10 per cage), while treatment (SI) mice were separated at the beginning of the SI paradigm. The SI mice were individually housed for 14 days in Plexiglas cages (26 × 20 × 14 cm) containing bedding and nesting material, with ad libitum access to food and water. All groups were maintained under identical environmental conditions (temperature, lighting, diet, and water). Body weight was recorded daily for SI mice and every three days for the controls to minimize handling stress. Behavioral testing during the isolation period included the elevated plus maze (day 4), open field test (day 7), and PhenoTyper^®^ assessment (days 8–10; EthoVision 3.0, Noldus IT, Wageningen, The Netherlands) for home-cage activity. Due to limited apparatus availability PhenoTyper^®^ testing was conducted over three consecutive days. One SI *Negr1^−/−^* male mouse was euthanized on day 8 after >20% body weight loss. On day 14, after a two-day rest period in their respective housing conditions, all mice were euthanized by decapitation for brain tissue collection, details of tissue collection is described in Section 2.6.

### 2.3. Elevated Plus Maze (EPM)

The elevated plus maze setup consisted of two open arms (measuring 17.5 × 5 cm) positioned opposite each other, lacking sidewalls, and two enclosed arms of the exact dimensions with sidewalls measuring 14 cm in height and an end wall. The maze was elevated to a height of 30 cm and situated in a room with a light intensity of 45 lx within the open arms. Mice were brought to the test room 60 min before the test for habituation. During the test, each animal was placed on the central platform of the maze, facing one of the open arms. After each mouse’s trial, the maze floor was cleaned with 70% ethanol and dried. A standard test duration of 5 min was maintained, with all sessions being video-recorded from above to cover the entire arena. The behavior parameters from the camera were assessed using Ethovision XT (Noldus Information Technology, Wageningen, The Netherlands), operated by the experimenter. An entry into an arm was considered only when all four limbs of the mouse were within the confines of that arm.

### 2.4. Open Field Test (OFT)

Mice were placed in the experimental room 60 min before the test for habituation. The locomotor activity of each mouse was assessed by placing them individually in photoelectric motility boxes (measuring 44.8 × 44.8 × 45 cm) for 30 min. The illumination level in the room was maintained at 450 lx throughout the experiment. These boxes were connected to a computer (TSE, Technical & Scientific Equipment GmbH, Berlin, Germany). Before each trial, the floor of the testing apparatus was cleaned using 70% ethanol and thoroughly dried. The system automatically recorded various parameters of the mice movement, including distance travelled, number of rearings, rearing frequency, corner visits, time spent, and distance covered within the central area of the box.

### 2.5. 24 h Monitoring in PhenoTyper^®^

The effect of social isolation on locomotor activity, reaction to a novel environment and anxiety-like behavior of each mouse was monitored for 24 h in PhenoTyper^®^ (EthoVision 3.0, Noldus Information Technology, Wageningen, The Netherlands). During the test, mice were individually housed in 30 cm × 30 cm × 35 cm Plexiglass cages with sawdust bedding. Mice had access to food and water throughout the testing period with a 12:12 h light and dark phase. Each cage had a top unit with an integrated infrared-sensitive camera and infrared LED lights for tracking during the dark phase. After 24 h of recording, system-generated data were used for analysis. For monitoring purposes, the arena was virtually divided into four zones: the whole arena, the center zone, the food and water zone, and the shelter zone. The distance travelled and time spent in each zone were taken into account for analysis and only the first 2 h of activity data were analyzed. The data were transformed (log2) to reduce skewness and normalize. Body weight, food and water were measured before introducing and after removing the mice from PhenoTyper^®^.

### 2.6. Brain Tissue Dissection and qPCR

**Figure 1:**
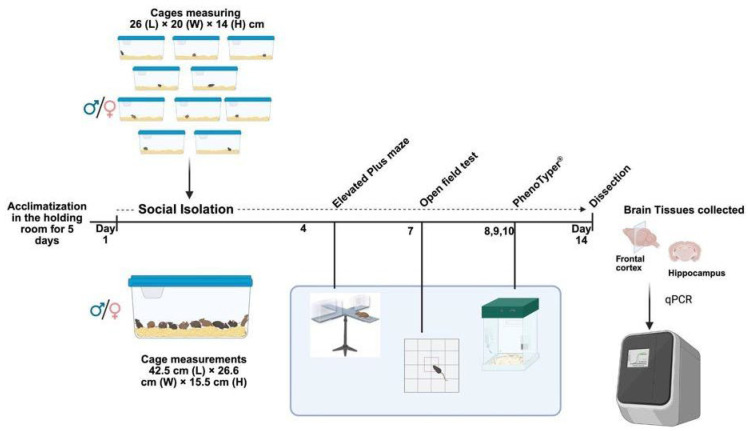
Graphical description of the experimental design. The illustration was made using BioRender (https://www.biorender.com) scientific image and illustration software.

On day 14, all mice were euthanized by decapitation, and brains were rapidly extracted. The prefrontal cortex and the whole hippocampus from both hemispheres were dissected according to coordinates from the mouse brain atlas [40]. For prefrontal cortex dissection, a coronal cut was made immediately posterior to the olfactory bulbs, followed by a second cut ~2 mm posterior to isolate the anterior cortical block. The hippocampi were then exposed by removing the overlying parietal cortex and gently rolled out intact from each hemisphere. All tissue samples were snap-frozen in liquid nitrogen and stored at −80 °C until RNA extraction for RT-qPCR analysis (Figure 1). Total RNA was extracted using the Trizol reagent (Invitrogen, Waltham, MA, USA), following the manufacturer’s guidelines. Subsequently, complementary DNA (cDNA) was synthesized from 1 µg of total RNA using the Firescript RT cDNA Synthesis Kit with Oligo(dT) and random primers (Solis BioDyne, Tartu, Estonia). Gene expression was quantified using a two-step RT-qPCR protocol. Expression of the following target genes was assessed: *Fgfr2*, *Cntn1*, *Gad1*, *Gad2*, *Pvalb*, *BDNF*, *TrkB (Ntrk2)*, *Tcf4-tot*, *Ntm1a*, *Ntm1b*, *Lsamp1a*, and *Lsamp1b*. *Negr1* expression was measured in Wt samples only, while for other *IgLON* genes, transcripts derived from the *Lsamp 1a*,*1b* and *Ntm1a*,*1b* promoters were quantified separately, following the promoter structure described by [40,41,42]. *Actb*, *Gapdh*, *Pgk1*, and *Hprt* were used as housekeeping reference genes.

RT-qPCR results were expressed on a linear scale as 2^−ΔCT^, following the method described in previous literature [43], where ΔCT denotes the difference in cycle threshold (CT) values between the target gene and the reference gene. Normalization of real-time RT-qPCR data was performed using the geometric mean of ΔCT values across multiple housekeeping genes, as described previously [44]. Four internal control genes—*Actb*, *Gapdh*, *Hprt*, and *Pgk1*—were used for this purpose. The geometric average of their ΔCT values was calculated, and the transformed (2^−ΔCT^) was used as the final expression levels. The primers used, along with their sequences and sources, are listed in Table 1.

### 2.7. Statistical Analysis

Statistical analysis was performed with GraphPad Prism 10.6.1. The data were tested for outliers using the ROUT method, and normality and lognormality were assessed using the Shapiro–Wilk test. Depending on the parameters, three-way ANOVA (factors: days, treatment and genotype) and two-way ANOVA (Factors: treatment and genotype), followed by a Bonferroni post hoc test were performed. For comparison of two groups (Supplementary Materials) depending on the data distribution, parametric *t*-test with Welch’s correction or a non-parametric Mann–Whitney *t*-test was performed to confirm significance. Results are presented as the mean ± SEM. A *p* < 0.05 was considered statistically significant. Significance levels in figures are indicated as follows: *p* < 0.05 (*), *p* < 0.01 (**), *p* < 0.001 (***), and *p* < 0.0001 (****) The symbol $ denotes the main effect of Genotype, # denotes the main effect of Treatment, and interaction effects (Treatment × Genotype) are indicated by X. The number of symbols consistently denotes the corresponding significance level throughout the manuscript. 

## 3. Results

### 3.1. Effect of Treatment and Genotype on Body Weight and Anxiety-like Behavior

**Figure 2:**
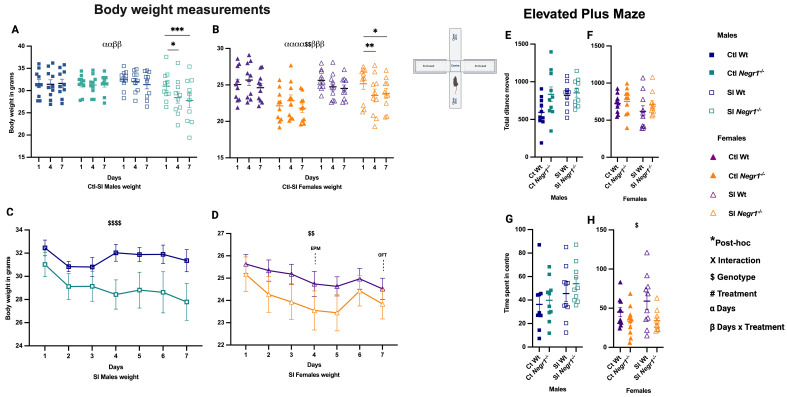
(**A**) Body weight on Day 1, Day 4, and Day 7 in males (n = 10). (**B**) Body weight on Day 1, Day 4, and Day 7 in females (n = 10). (**C**) Body weight dynamics of socially isolated (SI) males across 7 days (n = 10). (**D**) Body weight dynamics of socially isolated (SI) females across 7 days (n = 10). (**E**–**H**) Behavior in the 5 min elevated plus maze on Day 4 in Ctl and SI males and females. Body weight across time points (**A**,**B**) was analyzed by three-way repeated-measures ANOVA (factors: days, treatment, genotype) with Bonferroni post hoc tests. Body weight of SI groups (**C**,**D**) was analyzed by two-way ANOVA (factors: genotype, treatment) with Bonferroni post hoc tests. Elevated plus maze (**E**,**F**). Total distance moved (**G**,**H**). Time spent in center was analyzed by two-way ANOVA (factors: genotype, treatment) with Bonferroni post hoc tests. Parametric *t*-test with Welch’s correction was used to confirm significant effects between two groups. Data are presented as the mean ± SEM. Post hoc significance levels are indicated as * *p* < 0.05, ** *p* < 0.01, and *** *p* < 0.001, with the number of symbols indicating the corresponding significance level. The symbols $, $$ and $$$$ denote the main effect of Genotype at increasing significance levels. The symbol α indicates the main effect of Days, and β indicates the Days × Treatment interaction. Combined symbols (e.g., ααββ or αααα$$ββ) indicate that multiple effects—Days, Genotype, Treatment, and/or their interactions—are simultaneously significant. GraphPad Prism version 10.6.1 was used for data analysis, and the figure panel was created using BioRender scientific image and illustration software.

During the 14-day SI treatment, body weight for all mouse groups was measured on Day 1, Day 4, and Day 7. In males, repeated-measures analysis showed a main effect of days (F_2,18_ = 7.046; *p* = 0.006) and a days × treatment interaction (F_2,18_ = 7.319; *p* = 0.005). Post hoc comparisons indicated that SI *Negr1^−/−^* males had reduced body weight on Day 4 and Day 7 compared to Day 1 (Figure 2A; * *p* < 0.012 and *** *p* < 0.0005). In females, effects were more pronounced, with a main effect of days (F_2,18_ = 16.40; *p* < 0.0001), a genotype effect (F_1,9_ = 14.08; *p* = 0.005), and a days × treatment interaction (F_2,18_ = 13.80; *p* = 0.0002). Post hoc analysis showed reduced body weight on Day 4 compared to Day 1 (Figure 2B; ** *p* < 0.001), whereas by Day 7, SI *Negr1^−/−^* females showed partial recovery relative to Day 1 (Figure 2B; * *p* < 0.019). These results suggest that females were more resilient and regained weight more rapidly than males. Trajectories for SI Wt vs. SI *Negr1^−/−^* males and females are shown separately in Figure 2C,D. Significant genotype effects were detected in both sexes (males: F_6,132_ = 19.76; *p* < 0.0001; females: F_1,132_ = 7.32; *p* = 0.0077). On Day 4, EPM testing was performed. No significant differences in total distance moved were observed between groups in either sex (Figure 2E,F). However, time spent in the center of the arena (Figure 2G,H) indicated that *Negr1^−/−^* females spent less time in the center (Figure 2H) with a genotype effect (F_1,35_ = 6.340; *p* = 0.017), suggesting altered risk assessment or anxiety-like behavior. Additionally, an unpaired *t*-test with Welch’s correction showed that SI Wt males exhibited significantly higher total distance traveled compared to Ctl Wt males. These results, along with additional EPM parameters (distance moved in center, open and closed arms, time spent in arms, and head dips), are presented in Supplementary Figure S1.

### 3.2. Changes in Behavior During Social Isolation: Effect of Social Isolation on Locomotion, Exploratory Activity, and Anxiety-like Behavior

**Figure 3:**
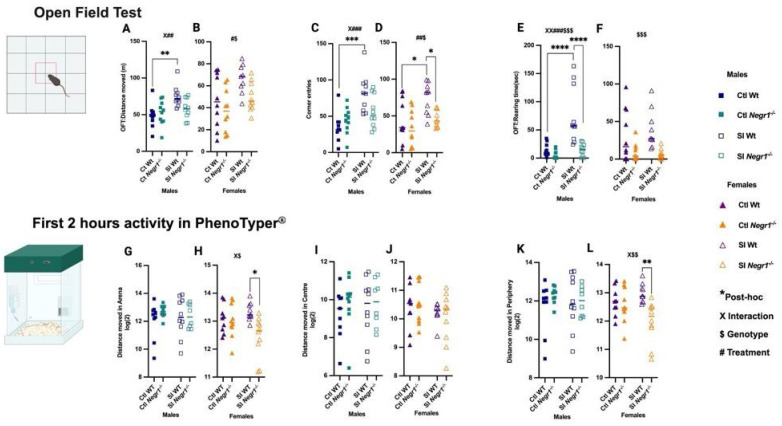
**Open field test (OFT)**: (**A**,**B**) Distance moved in arena; (**C**,**D**) Corner entries, (**E**,**F**) Rearing time (Males, n = 10; females, n = 10). **PhenoTyper^®^ (First 2 h)** (**G**,**H**); Distance moved in Arena; (**I**,**J**) Distance moved in Center; (**K**,**L**) Distance moved in periphery by Ctl and SI male and female mice [Males (n = 10), Females (n = 10)]. Two-way ANOVA with Bonferroni’s post hoc test (factors: genotype, treatment) was performed. A parametric *t*-test with Welch’s correction was used to confirm significant effects between two groups. All data are represented as the mean ± SEM and post hoc significance is presented as: * *p* < 0.05,** *p* < 0.01, *** *p* < 0.001, **** *p* < 0.0001. The number of other symbols consistently denotes the corresponding significance level. The symbol # indicates a main effect of Treatment, while $ indicates a main effect of Genotype. Interaction effects (Treatment × Genotype) are indicated by X. Combined symbols (e.g., X##, X###, XX###$$$, #$, ##$, X$, X$$, $$$) denote that multiple effects—treatment, genotype, and/or interaction—are simultaneously significant. GraphPad Prism version 10.6.1 was used for data analysis, and the figure panel was created using BioRender: Scientific image and illustration software.

On Day 7, OFT revealed sex-specific effects of genotype and SI treatment. In males, SI increased the total distance moved in SI Wt mice when compared against Ctl Wt (Figure 3A; ** *p* < 0.0026), with a genotype × treatment interaction (F_1,36_ = 4.643; *p* = 0.038) and a treatment effect (F_1,36_ = 11.01; *p* = 0.002). Females also showed elevated locomotion (Figure 3B) supported by a treatment effect (F_1,35_ = 7.393; *p* = 0.01) and a genotype effect (F_1,35_ = 4.851; *p* = 0.03), although no post hoc differences were detected. SI Wt males made more corner entries than Ctl Wt (Figure 3C; *** *p* < 0.0001), consistent with a genotype × treatment interaction (F_1,36_ = 6.233; *p* = 0.017) and a strong treatment effect (F_1,36_ = 18.84; *p* = 0.0001). SI Wt females similarly exhibited increased corner visits (Figure 3D; * *p* < 0.0114, * *p* < 0.0131) with a treatment effect (F_1,35_ = 7.583; *p* = 0.0093) and genotype effect (F_1,35_ = 7.138; *p* = 0.0114) compared to Ctl Wt and SI *Negr1^−/−^*. Social isolation robustly increased rearing time in SI Wt males (Figure 3E; **** *p* < 0.0001), supported by an interaction (F_1,36_ = 9.563; *p* = 0.0038), a strong treatment effect (F_1,36_ = 16.84; *p* = 0.0002), and a genotype effect (F_1,36_ = 16.84; *p* = 0.0002) when compared against Ctl Wt and SI *Negr1^−/−^* whereas females showed a genotype effect (F_1,35_ = 15.00; *p* = 0.0005) (Figure 3F). Parametric *t*-test with Welch’s correction further revealed higher rearing time in SI Wt females compared to SI *Negr1^−/−^* females (Supplementary Figure S2). Together, OFT results indicate that SI Wt mice showed stress-induced hyperactivity and decreased anxiety-like behavior compared to Ctl Wt and SI *Negr1^−/−^* mice. Additional OFT parameters are presented in Supplementary Figure S2.

For continuous activity monitoring, mice were recorded for 24 h in the PhenoTyper^®^, and the first 2 h were analyzed. In males, total distance moved did not differ across groups (Figure 3G). In females, an interaction (F_1,34_ = 5.017; *p* = 0.0317) and genotype effect (F_1,34_ = 6.618; *p* = 0.0146) were observed, with SI Wt females showing higher activity than SI *Negr1^−/−^* females (Figure 3H; * *p* < 0.0103). Distance moved in the center did not differ in either sex (Figure 3I,J). Distance moved in the periphery showed no differences in males (Figure 3K). While females displayed a genotype × treatment interaction (F_1,34_ = 5.769; *p* = 0.0219) and genotype effect (F_1,34_ = 9.024; *p* = 0.0050), SI Wt females moved more in the periphery than SI *Negr1^−/−^* females (Figure 3L; ** *p* < 0.0032). Additional shelter-related behaviors and zone allocation (arena, center, periphery) are shown in Supplementary Figure S3. Body weight, food intake, and water consumption before and after PhenoTyper^®^ monitoring are provided in Supplementary Figure S4. Collectively, these results demonstrate that SI and *Negr1* deficiency interact to shape locomotor and exploratory behaviors, with stronger SI-induced hyperactivity in males in the OFT and more pronounced exploratory effects in SI females in the PhenoTyper^®^.

### 3.3. Dysregulation in the GABAergic Markers Following Social Isolation

**Figure 4:**
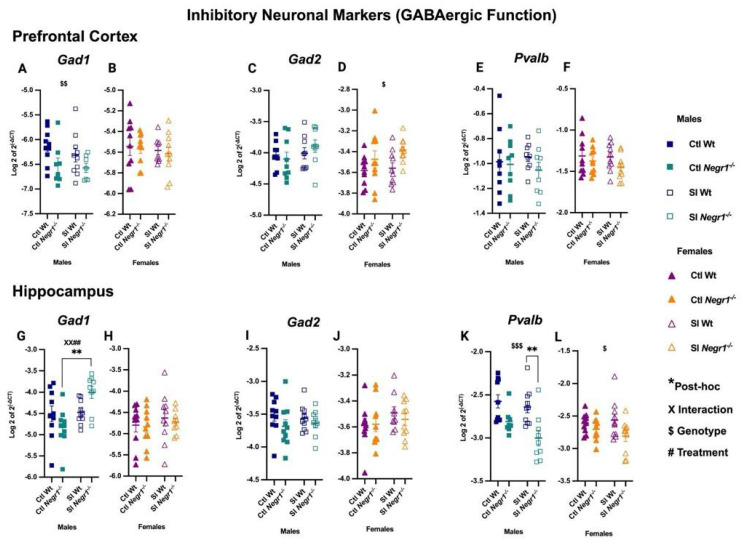
GABAergic marker gene expression in the prefrontal cortex: (**A**,**B**) *Gad1*, (**C**,**D**) *Gad2*, (**E**,**F**) *Pvalb*; and in the hippocampus: (**G**,**H**) *Gad1*, (**I**,**J**) *Gad2*, (**K**,**L**) *Pvalb* [Males (n = 10), Females (n = 10)]. Two-way ANOVA (factors: genotype, treatment) with Bonferroni post hoc tests was performed. Data are presented as the mean ± SEM. Post hoc significance is indicated as:, ** *p* < 0.01. $, $$, and $$$ denote the main effect of Genotype at increasing significance levels; # and X denote the Treatment and Interaction between Genotype and treatment. Combined symbols (XX##) indicate multiple simultaneously significant effects. GraphPad Prism version 10.6.1 was used for data analysis, and the figure panel was created using BioRender scientific image and illustration software.

RT-qPCR analysis revealed dysregulation of GABAergic markers in the prefrontal cortex. In males, *Gad1* expression showed a significant genotype effect (F_1,30_ = 10.29; *p* = 0.0032) (Figure 4A), whereas no changes were observed in females (Figure 4B). Conversely, *Gad2* expression showed a genotype effect in females (F_1,36_ = 6.122; *p* = 0.0182) (Figure 4D), while males showed no difference (Figure 4C). The interneuron marker *Pvalb* did not differ between groups in either sex (Figure 4E,F). In the hippocampus, both treatment and genotype effects were evident. *Gad1* expression was significantly increased in SI *Negr1^−/−^* males compared to Ctl *Negr1^−/−^* males, supported by a genotype × treatment interaction (F_1,35_ = 7.540; *p* = 0.0095) and a treatment effect (F_1,35_ = 8.920; *p* = 0.0051) (Figure 4G; ** *p* < 0.0019). No such changes were observed in females (Figure 4H). *Gad2* expression remained unchanged across groups in both sexes (Figure 4I,J). In contrast, *Pvalb* was reduced in SI Wt males compared to SI *Negr1^−/−^* males (F_1,33_ = 16.39; *p* = 0.0003) (Figure 4K; ** *p* < 0.0074). A similar, albeit milder, genotype effect was observed in females (F_1,36_ = 5.878; *p* = 0.0205) (Figure 4L). Together, these findings show that *Gad1*, *Gad2*, and *Pvalb* exhibit region-specific and sex-dependent regulation, which may contribute to the behavioral differences observed between males and females. Analyses combining data from both sexes are provided in Supplementary Figure S6. Furthermore, to investigate *Pvalb* significance between SI Wt and SI *Negr1^−/−^* when data were combined, a parametric *t*-test with Welch’s correction was performed. The related graph can be found in Supplementary Figure S9.

### 3.4. Change in Neurotrophic Signalling-Related Genes Post Social Isolation

**Figure 5:**
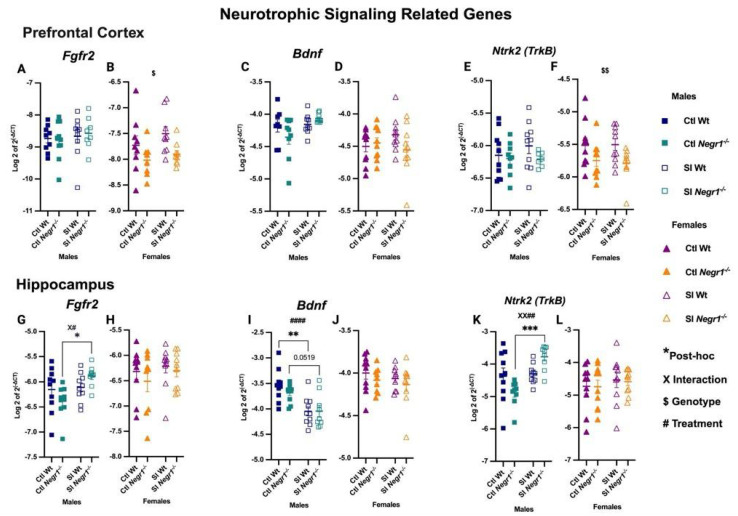
Gene expression of neurotrophic signaling-related transcripts in the prefrontal cortex: (**A**,**B**) *Fgfr2*, (**C**,**D**) *Bdnf*, (**E**,**F**) *Ntrk2*; and in the hippocampus: (**G**,**H**) *Fgfr2*, (**I**,**J**) *Bdnf*, (**K**,**L**) *Ntrk2* [Males (n = 10), Females (n = 10)]. Two-way ANOVA (factors: genotype, treatment) with Bonferroni post hoc tests was performed. Data are presented as the mean ± SEM. Post hoc significance is indicated as: * *p* < 0.05, ** *p* < 0.01, *** *p* < 0.001. $ and $$ denote the main effect of Genotype (*p* < 0.05, *p* < 0.01) and #### indicate a main effect of Treatment (*p* < 0.0001), while XX## indicates a combined significant effect involving both Interaction (X) and Treatment (#). GraphPad Prism version 10.6.1 was used for data analysis, and the figure panel was created using BioRender scientific image and illustration software.

In the prefrontal cortex, *Fgfr2* expression did not differ in males (Figure 5A), whereas females showed a genotype effect (F_1,31_ = 6.74; *p* = 0.0142), with lower expression in *Negr1^−/−^* groups compared to Wt (Figure 5B). *Bdnf* expression remained unchanged in both sexes (Figure 5C,D). In contrast, *Ntrk2* (TrkB) showed a genotype-dependent increase in females (F_1,35_ = 7.599; *p* = 0.0092) (Figure 5F), while no differences were observed in males (Figure 5E). In the hippocampus, *Fgfr2* was elevated in SI *Negr1^−/−^* males compared to Ctl *Negr1^−/−^* males, supported by a genotype × treatment interaction (F_1,35_ = 4.749; *p* = 0.0360) and a treatment effect (F_1,35_ = 6.756; *p* = 0.0136) (Figure 5G; * *p* < 0.0121). No changes were observed in females (Figure 5H). *Bdnf* expression showed a strong treatment effect in males (F_1,35_ = 23.15; *p* < 0.0001), with SI Wt males exhibiting reduced expression compared to Ctl Wt, and SI *Negr1^−/−^* males showing a trend toward reduction relative to Ctl *Negr1^−/−^* (Figure 5I; ** *p* < 0.0017; *p* = 0.0519) while females showed no significant effects (Figure 5J). In contrast to *Bdnf*, *Ntrk2* expression increased in SI *Negr1^−/−^* males compared to Ctl *Negr1^−/−^* males, with a genotype × treatment interaction (F_1,34_ = 10.09; *p* = 0.0032) and a treatment effect (F_1,34_ = 12.81; *p* = 0.0011) (Figure 5K; *** *p* < 0.0003). No such changes were observed in females (Figure 5L). Combined-sex analyses are available in Supplementary Figure S7. Furthermore to investigate significance between certain groups when data were combined, a parametric *t*-test with Welch’s correction was performed when data were normal and a non-parametric Mann–Whitney *t*-test was performed when the data were not normally distributed. A related graph can be found in Supplementary Figure S9.

### 3.5. Expression of Cell Adhesion Molecule Transcripts and Related Interactors

**Figure 6:**
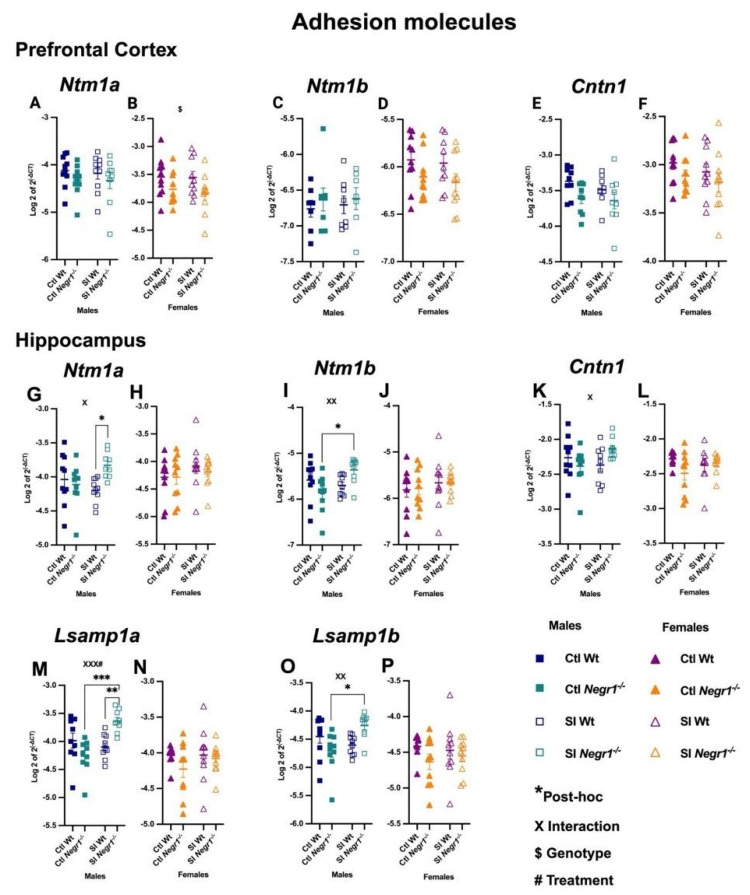
Neural cell adhesion transcripts in the prefrontal cortex (**A**,**B**) *Ntm1a*, (**C**,**D**) *Ntm1b*, (**E**,**F**) *Cntn1*, in hippocampus (**G**,**H**) *Ntm1a*, (**I**,**J**) *Ntm1b*, (**K**,**L**) *Cntn1*, (**M**,**N**) *Lsamp1a*, (**O**,**P**) *Lsamp1b* [Males (n = 10), Females (n = 10)]. Two-way ANOVA with Bonferroni’s post hoc test (factors: genotype, treatment) was performed. An unpaired *t*-test with Welch’s correction was used to confirm significant effects in *Negr1* expression presented (Supplementary Figure S8). All data are represented as the mean ± SEM and post hoc significance is presented as: * *p* < 0.05, ** *p* < 0.01, *** *p* < 0.001. $ denotes the main effect of Genotype (*p* < 0.05), while Interaction effects (Treatment × Genotype) are indicated by X and XX (*p* < 0.05, *p* < 0.01) XXX# indicates a combined significant effect involving both Interaction (X) and Treatment (#). GraphPad Prism version 10.6.1 was used for data analysis, and the figure panel was created using BioRender scientific image and illustration software.

In the prefrontal cortex, cell adhesion molecule expression showed minimal changes. *Ntm1a* displayed no differences in males, whereas females showed a genotype effect (F_1,36_ = 5.826; *p* = 0.0210) (Figure 6A,B). *Ntm1b* showed no differences in either sex (Figure 6C,D), and *Cntn1* expression remained stable across all groups (Figure 6E,F). In contrast, several significant changes were observed in the hippocampus, particularly in males. *Ntm1a* was increased in SI *Negr1^−/−^* males compared to SI Wt, with a genotype × treatment interaction (F_1,35_ = 6.137; *p* = 0.0182) (Figure 6G; * *p* < 0.0406), whereas females showed no differences (Figure 6H). *Ntm1b* was similarly elevated in the hippocampus of SI *Negr1^−/−^* males compared to Ctl *Negr1^−/−^* males, supported by an interaction (F_1,35_ = 7.824; *p* = 0.0083) (Figure 6I; * *p* < 0.0193), with no change in females (Figure 6J). *Cntn1* expression also showed a genotype × treatment interaction in the hippocampal area of males (F_1,34_ = 4.513; *p* = 0.0410), however, no post hoc effect was seen (Figure 6K), whereas females again showed no differences (Figure 6L). These male-specific increases extended to *Lsamp* transcripts. *Lsamp1a* was elevated in SI *Negr1^−/−^* males relative to both SI Wt and Ctl *Negr1^−/−^*, with a strong interaction (F_1,33_ = 14.95; *p* = 0.0005) and treatment effect (F_1,33_ = 7.288; *p* = 0.0109) (Figure 6M; *** *p* < 0.0004, ** *p* < 0.0088) while no changes were observed in females (Figure 6N). *Lsamp1b* was likewise increased in SI *Negr1^−/−^* males compared to Ctl *Negr1^−/−^* males (F_1,35_ = 10.44; *p* = 0.0027) (Figure 6O; * *p* < 0.0111), with no changes in females (Figure 6P). Additional gene expression data—including *Negr1* and other markers—are provided in Supplementary Figure S5. Combined-sex analyses of IgLONs and *Cntn1* are presented in Supplementary Figure S8.

## 4. Discussion

In this study, we examined the behavioral and molecular consequences of 14 days of social isolation stress in Wt and *Negr1^−/−^* mice of both sexes. Social isolation is a well-established model of psychosocial stress that induces behavioral and neurobiological changes relevant to psychiatric disorders [55,56]. Given that *Negr1* is implicated in neurodevelopment, synaptic plasticity, and genetic risk for neuropsychiatric disease [21,57,58,59], we investigated how its loss modulates susceptibility to environmental stress. This is the first study to assess the effects of social isolation specifically in *Negr1^−/−^* mice, focusing on genes linked to GABAergic transmission, neurotrophic signaling, and cell adhesion. Prior research shows that social isolation alters sociability, aggression, PV^+^ interneurons, *Gad1/2* expression, and *Bdnf–TrkB* signaling [60,61]. Consistently, *Gad1* haploinsufficiency leads to social and affective deficits and reductions in PV^+^ interneurons [61,62]. Neurotrophic genes such as *Fgfr2*, *Bdnf*, and *TrkB* are key convergence points for Negr1-related signaling and stress responses, and are disrupted by social isolation [63,64]. These interconnected pathways provide a mechanistic framework for understanding how Negr1 deficiency may alter GABAergic function under stress.

*Negr1^−/−^* mice do not exhibit a consistent baseline behavioral phenotype: one study reported no changes in the elevated plus maze or open field [21], whereas another found increased center activity in the open field [22]. This variability led us to hypothesize that social isolation may unmask or amplify subtle genotype-dependent differences in anxiety- and exploration-related behaviors. Given our previous findings of marked sex differences in stress adaptation [65], both sexes were included in the present study. By integrating behavioral assessments with gene expression analyses in the prefrontal cortex and hippocampus, we identified genotype and sex-specific interactions between Negr1 deficiency and social isolation, providing new insights into molecular pathways underlying stress-related behavioral phenotypes.

### 4.1. Behavioral and Body Weight Effects of Social Isolation Stress

Body weight regulation during social isolation showed clear sex- and genotype-dependent effects. SI *Negr1^−/−^* males exhibited sustained weight loss across isolation days, whereas Wt males recovered by Day 4, suggesting impaired metabolic or stress adaptation in *Negr1* deficiency, consistent with altered stress reactivity in vulnerable genotypes [66]. In females, both genotypes lost weight, but *Negr1^−/−^* female mice showed a milder decline that stabilized by Day 7, indicating partial resilience compared to males. Interestingly, in our earlier studies with *Lsamp* knockout males, body weight remained stable during isolation, showing resistance to isolation-induced weight loss [67], highlighting that different IgLON family members may differentially modulate stress-related metabolic adaptation, *Negr1* deficiency enhancing, and *Lsamp* deficiency buffering, the physiological impact of social isolation.

Behavioral assays revealed that social isolation increased locomotor activity in SI Wt males, but this hyperactivity was attenuated in *Negr1*^−/−^ males. SI Wt males exhibited greater exploratory drive and reduced anxiety-like behavior, a typical short-term adaptive response to moderate isolation stress. In females, *Negr1*^−/−^ mice spent less time in the center zone in EPM, suggesting heightened anxiety-like behavior or reduced exploratory motivation. Similar patterns emerged in the open-field and PhenoTyper assays: stress-induced hyperactivity was evident in SI Wt but not SI *Negr1^−/−^* mice, particularly in males. SI Wt males were markedly more active, showing increased locomotion, corner exploration, and rearing behavior. Conversely, SI *Negr1^−/−^* mice lacked this exploratory activation, especially females, whose peripheral activity and exploration were lowest among all groups. These results suggest that *Negr1* deletion diminishes the capacity to engage adaptive exploratory or coping behaviors under stress, consistent with reduced behavioral flexibility and emotional resilience. Such rigidity has been linked to maladaptive stress responses and emotional dysregulation in models of psychiatric disorders [66,67].

Overall, *Negr1* deficiency disrupted typical behavioral adaptations to social stress, with males showing impaired physiological recovery and females displaying enhanced anxiety-like traits. These effects are consistent with well-documented sex-dependent differences in stress reactivity and coping strategies in rodents [68,69].

### 4.2. GABAergic Regulation and Parvalbumin Interneurons

At the molecular level, *Negr1* deficiency produced distinct GABAergic alterations across brain regions and sexes. In the prefrontal cortex, *Gad1* downregulation in *Negr1*^−/−^ males suggests a reduction in basal GABA synthesis capacity [70], whereas *Gad2* upregulation in the SI females *Negr1*^−/−^ group (Supplementary Figure S5) might represent a compensatory mechanism to maintain inhibitory tone. These differential responses indicate that sex-specific transcriptional regulation contributes to how inhibitory systems adapt to social stress in the absence of Negr1.

In the hippocampus, *Gad1* expression was upregulated in SI *Negr1*^−/−^ males, suggesting stress-induced transcriptional plasticity within inhibitory networks. This increase, however, may not translate into effective synaptic inhibition, given the concurrent reduction in *Pvalb* expression in the same group. Parvalbumin-positive interneurons are critical for temporal precision and synchronization of inhibitory signaling; thus, their downregulation implies compromised excitatory–inhibitory (E/I) balance and impaired oscillatory regulation. The milder *Pvalb* decrease in *Negr1*^−/−^ females points to partial resilience of inhibitory circuits, aligning with their subtler behavioral alterations.

Deficits in Pvalb interneurons are a well-recognized hallmark of neurodevelopmental and affective disorders, including schizophrenia and major depression, where E/I imbalance leads to cortical desynchronization and cognitive dysfunction [26,27,70,71]. The present findings extend this concept by linking Negr1-mediated cell adhesion to the stability of Pvalb interneurons, suggesting that *Negr1* may influence molecular pathways critical for maintaining inhibitory circuit integrity. The combined changes in *Gad1*, *Gad2*, and *Pvalb* indicate that *Negr1* deficiency disrupts GABAergic transcriptional homeostasis, rendering neuronal networks less capable of adapting to stress-induced excitation—effects that were particularly pronounced in Wt males.

### 4.3. Gene Expression of Neurotrophic Signalling

Social isolation produced strong, sex-specific effects on neurotrophic signaling. In the hippocampus of males, *Bdnf* expression was downregulated, whereas its receptor *Ntrk2* (*TrkB*) was upregulated, suggesting a compensatory feedback response to diminished neurotrophin availability. Such opposite regulation of ligand and receptor may reflect an adaptive mechanism aimed at maintaining synaptic responsiveness and neuronal stability under stress [72,73]. The lack of similar changes in females indicates sex-dependent differences in neurotrophic adaptability to social isolation [67,68].

*Fgfr2* expression showed opposite effects across brain regions and sexes—elevated in the hippocampus of stressed *Negr1*^−/−^ males but reduced in *Negr1*^−/−^ females in the prefrontal cortex—suggesting to sex-specific modulation of growth factor signaling [58]. *Tcf4*, a transcription factor linked to neuronal differentiation and psychiatric risk, was downregulated in the prefrontal cortex of *Negr1*^−/−^ female mice but showed increased hippocampal expression under isolation in *Negr1*^−/−^ males (Supplementary Figure S5). It has previously been shown that chronic social stress combined with altered *Tcf4* induces cognitive dysfunction [74]. Together, these findings suggest that *Negr1* interacts with multiple neurotrophic pathways, and the balance between *Bdnf-TrkB* signaling and downstream regulators, such as *Fgfr2* and *Tcf4*, may critically influence how male and female brains adapt to social stress.

### 4.4. Gene Expression of Neural Adhesion Molecules

Among neural adhesion molecules, *Cntn1* showed a strong genotype and stress-dependent regulation in the hippocampus in males (Supplementary Figure S5). As a known physical interaction partner of *Negr1* [25,31], Cntn1, an immunoglobulin superfamily member, has been previously shown to increase in the hippocampus following chronic unpredictable stress, where its upregulation correlates with anxiety- and depression-like behaviors [46]. In line with the stress sensitivity of this pathway, we observed a genotype × treatment interaction in males: while baseline Cntn1 expression was higher in Wt mice, SI selectively increased hippocampal Cntn1 in *Negr1^−^/^−^* males, reversing the baseline pattern. Females again showed no changes. This male-specific shift suggests that the absence of Negr1 alters how hippocampal adhesion-related signaling responds to stress, potentially heightening vulnerability to maladaptive circuit remodeling under adverse conditions.

Other IgLON family members also showed marked region and sex-dependent expression patterns. In the prefrontal cortex, *Ntm1a* was downregulated in *Negr1*^−/−^ females with a genotype effect, whereas in the hippocampus, both *Ntm* and *Lsamp* isoforms exhibited strong genotype x treatment interactions in males, *Ntm1a/1b and Lsamp1a/1b* transcripts were strongly upregulated in SI *Negr1^−/−^* males compared to SI Wt (Supplementary Figure S5). This result is in line with our previous studies showing that in Wt mice, SI does not induce *Lsamp1a/1b* transcript expression [75]. These findings suggest that social stress unmasks latent regulatory mechanisms within the IgLON family, particularly in males, supporting our previous results demonstrating that particularly Lsamp, but also Ntm, regulate social and emotional behavior in mice [42,76,77]. Notably, *Negr1* RNA itself was not affected by isolation (Supplementary Figure S5), indicating that stress susceptibility likely arises from altered downstream adhesion networks rather than the direct regulation of *Negr1*.

Given the critical role of IgLON proteins in synapse formation and psychiatric vulnerability [31,41,73,78] and their coordinated expression with *Negr1*, these suggest a compensatory interplay maintaining synaptic cohesion. Such adaptive transcriptional activation may help stabilize connectivity in limbic circuits compromised by stress or genetic loss of adhesion components and reflects a multi-level adaptive process aimed at preserving neuronal communication and stress resilience.

## 5. Conclusions

In summary, we demonstrate that *Negr1* deficiency interacts with social isolation stress to produce sex-specific alterations in behavior and gene expression. Male *Negr1*^−/−^ mice showed impaired adaptation in weight regulation and locomotor activity, while *Negr1*^−/−^ females displayed heightened anxiety-like behavior and reduced stress-induced hyperactivity. At the molecular level, *Negr1* deficiency disrupted inhibitory interneuron markers, neurotrophic signaling, and neural adhesion molecules, with hippocampal expression patterns strongly shaped by genotype × treatment interactions. These findings highlight *Negr1* as a key modulator of stress susceptibility, bridging environmental stress exposure with molecular networks implicated in psychiatric disorders. Our results support the hypothesis that genetic variation in *Negr1* may contribute to differential vulnerability to psychosocial stress and related psychopathologies [12,56].

## 6. Limitations

Although this study integrates behavioral and molecular analyses, several limitations should be acknowledged. First, the weighing of control (group-housed) animals was performed less frequently to minimize handling-related stress. While daily weighing would have provided more detailed body weight dynamics, reduced handling ensured that control mice remained free from additional experimental interference. Second, due to limited access to the PhenoTyper^®^ apparatus and the large cohort size, testing was distributed over three consecutive days. To minimize variability between sessions, only the first two hours of data were analyzed for consistency. Third, the use of broadly expressed housekeeping genes for qPCR normalization may not have fully accounted for cell–type–specific expression differences, particularly for genes restricted to small neuronal subpopulations. To reduce this bias, we used the geometric mean of four reference genes (Hprt, Gapdh, Pgk1, and Actb), representing diverse cellular functions and expression levels. This approach minimizes the influence of variability in any single reference gene and provides a more stable normalization factor. Although minor effects related to cellular composition cannot be excluded, they are unlikely to affect the main conclusions. Finally, protein-level validation and immunohistochemical mapping of Pvalb and Negr1 expression are needed to confirm the cellular localization of transcriptional changes. Future studies should also extend molecular analyses beyond the prefrontal cortex and hippocampus to include the amygdala and hypothalamus, and integrate transcriptomic, proteomic, and imaging approaches for a systems-level understanding of Negr1’s role in stress-related neural circuits.

## Figures and Tables

**Table 1:** List of primers used for gene expression in the current study.

Target Genes	Primer Sequence	Amplicon Size	Sources
*Fgfr2*	F_TGCACGCAGGATGGACCTCTCT R_TGCTCCTCGGGGACACGGTTAA	131 bp	[45]
*Cntn1*	F_CGCGTTTCAAGTCAAAGTGA R_TTTGACCCCTACCTCTGTGG	121 bp	[46]
*Gad 1*	F_ATGATACTTGGTGTGGCGTAG R_GACTCTTCTCTTCCAGGCTATTG	99 bp	[47]
*Gad 2*	F_CATTGATAAGTGTTTGGAGCTAGCA R_GTGCGCAAACTAGGAGGTACAA	135 bp	[48]
*Pvalb*	F_TTCTGAAGGGCTTCTCCTCA R_TTCTTCAACCCCAATCTTGC	107 bp	[49]
*Bdnf*	F_TGGCTGACACTTTTGAGCAC R_AAGTGTACAAGTCCGCGTCC	99 bp	[50]
*TrkB(Ntrk2)*	F_AAGGACTTTCATCGGGAAGCTG R_TCGCCCTCCACACAGACAC	86 bp	[51]
*Tcf4-tot*	F_GACCACACGAACAACAGCTT R_TCTTCGATTCGGCTTTGCAG	161 bp	[52] based on human primers
*Negr1*	F_TGCTCGAACCAGTGGCTGGC R_CCCTTTGATGCTCCATCTTCCA	161 bp	[41]
*Ntm1a*	F_CTGGCGGCTCTGTGCCTCT R_GGTGACTCGGTTGTCAATTGTG	135 bp	[41]
*Ntm 1b*	F_CTCTCAGGCTGCTATTCCTTGTA R_GGTGACTCGGTTGTCAATTGTG	140 bp	[41]
*Lsamp 1a*	F_GCATTTTGGAACCAGCCTCCTG R_TTCTTGTCTTCTACCACACACCTG	155 bp	[53]
*Lsamp 1b*	F_CGATCGGAAACAGTTGCCGC R_TTCTTGTCTTCTACCACACACCTG	156 bp	[53]
*Pgk1*	F_CGTGATGAGGGTGGACTT R_TGGAACAGCAGCCTTGAT	79 bp	[54]
*Gapdh*	F_ACAATGAATACGGCTACAG R_GGTCCAGGGTTTCTTACT	78 bp	[54]
*Hprt*	F_GCAGTACAGCCCCAAAATGG R_AACAAAGTCTGGCCTGTATCCAA	85 bp	[41]
*Act B*	F_ACCATGTACCCAGGCATTGC R_AGCCACCGATCCACACAGAG	121 bp	[28]

## Data Availability

The raw data supporting the conclusions of this article will be made available by the authors on request.

## Supplementary Materials

The following supporting information can be downloaded at: https://www.mdpi.com/article/10.3390/brainsci15121286/s1, Figure S1: Activity in Elevated plus maze (EPM) (A,B) Distance moved in centre (C,D) Distance moved in closed arms (E,F) Distance moved in open arms (G,H) Time spent in closed arms (I,J) Time spent in open arms (K,L) Number of head dips. [Males (n = 10), Females (n = 10)]. Data were analysed by two-way ANOVA (factors: genotype, treatment) with Bonferroni post hoc tests. (M) Total distance moved by Ctl and SI Wts, a parametric *t*-test with Welch’s correction was used to confirm significant effects between the two groups. All data are represented as mean ± SEM and posthoc significance are presented as: * *p* < 0.01, ** *p* < 0.001, *** *p* < 0.0001. GraphPad Prism version 10.6.1 was used for data analysis, and the figure panel was created using BioRender: Scientific image and illustration software. Figure S2: Activities in Open field test (OFT) (A,B) Distance moved in corner (C,D) Time spent in corner (E,F) Number of rearings (G,H) Time spent in centre (I,J) Time spent in periphery (K,L) Clockwise and counter-clockwise rotations (Rotation CW+CCW) (M,N) Faecal pellet count during OFT. [Males (n = 10), Females (n = 10)]. Data were analysed by two-way ANOVA (factors: genotype, treatment) with Bonferroni post hoc tests. (O) Rearing time by SI Wt and SI Negr1^-/-^ females, a parametric *t*-test with Welch’s correction was used to confirm significant effects between the two groups. All data are represented as mean ± SEM and post-hoc significance are presented as: * *p* < 0.01, ** *p* < 0.001, *** *p* < 0.0001. GraphPad Prism version 10.6.1 was used for data analysis, and the figure panel was created using. BioRender: Scientific image and illustration software. Figure S3: Activities in shelter and time spent in other arenas. (A,B) Distance moved in shelter (C,D) Time spent in shelter (E,F) Time spent in arena (G,H) Time spent in centre (I,J) Time spent in periphery. [Males (n = 10), Females (n = 10)]. Data were analysed by two-way ANOVA (factors: genotype, treatment) with Bonferroni post hoc tests. All data are represented as mean ± SEM and posthoc significance are presented as: * *p* < 0.01, ** *p* < 0.001, *** *p* < 0.0001. GraphPad Prism version 10.6.1 was used for data analysis, and the figure panel was created using BioRender: Scientific image and illustration software. Figure S4: (A,B) Change in body weight(Δ Body weight) is shown in grams (C,D) Food consumed during 24 h in PhenoTyper^®^ (E,F) Water consumed during 24 h in PhenoTyper^®^. [Males (n = 10), Females (n = 10)]. Data were analysed by two-way ANOVA (factors: genotype, treatment) with Bonferroni post hoc tests. All data are represented as mean ± SEM and post-hoc significance are presented as: * *p* < 0.01, ** *p* < 0.001, *** *p* < 0.0001. GraphPad Prism version 10.6.1 was used for data analysis, and the figure panel was created using BioRender: Scientific image and illustration software. Figure S5: Gene expression in the prefrontal cortex (A,B) Tcf4 (C,D) Lsamp1a (E,F) Lsamp1b. Gene expression in the hippocampus (I,J) Tcf4 [Males (n = 10), Females (n = 10)]. Data were analysed by two-way ANOVA factors: genotype, treatment) with Bonferroni post hoc tests. In the prefrontal cortex (G,H) Negr1 (I) Gad2 and in the hippocampus (L,M) Negr1, (N) Tcf4, (O) Cntn1, (P) Ntm1b, (Q) Lsamp1b, a parametric *t*-test with Welch’s correction was used to confirm significance between two groups when the data were normally distributed, or else a non-parametric Mann-Whitney ttest was performed. All data are represented as mean ± SEM, and post-hoc significance is presented as: * *p* < 0.01, ** *p* < 0.001, *** *p* < 0.0001. GraphPad Prism version 10.6.1 was used for data analysis, and the figure panel was created using BioRender, a scientific image and illustration software. Figure S6: From the gene expression studies, data from both sexes were combined to get an overview. In the frontal cortex (A) Gad1 (B) GAD2 (C) Pvalb, in the hippocampus (D) Gad1 (E) Gad2 (F) Pvalb. [Males (n = 10), Females (n = 10)]. Data were analysed by two-way ANOVA (factors: genotype, treatment) with Bonferroni post hoc tests. All data are represented as mean ± SEM and posthoc significance are presented as: * *p* < 0.01, ** *p* < 0.001, *** *p* < 0.0001. GraphPad Prism version 10.6.1 was used for data analysis, and the figure panel was created using BioRender: Scientific image and illustration software. Figure S7: Expression of neurotropic signalling molecules, data from both sexes were combined to get an overview. In the frontal cortex (A) Fgfr2 (B) Bdnf (C) Ntrk2 (D) Tcf4. In the hippocampus (D) Fgfr2 (E) Bdnf (F) Ntrk2 (H) Tcf4 (Males, n = 10, females, n = 10). Data were analysed by two-way ANOVA (factors: genotype, treatment) with Bonferroni post hoc tests. All data are represented as mean ± SEM and post-hoc significance are presented as: * *p* < 0.01, ** *p* < 0.001, *** *p* < 0.0001. GraphPad Prism version 10.6.1 was used for data analysis, and the figure panel was created using BioRender: Scientific image and illustration software. Figure S8: Expression of IgLONs and Cntn1 when both sexes are combined. In the frontal cortex (A) Ntm1a (B) Ntm1b (C) Lsamp1a (D) Lsamp1b (E) Cntn1. In the hippocampus (G) Ntm1a (H) Ntm1b (I) Lsamp1a (J) Lsamp1b (K) Cntn1. (Males, n = 10, females, n = 10). Data were analysed by two-way ANOVA (factors: genotype, treatment) with Bonferroni post hoc tests. (F,L) Negr1, a parametric *t*-test with Welch’s correction was used to confirm significant effects between the Wts. All data are represented as mean ± SEM and post-hoc significance are presented as: * *p* < 0.01, ** *p* < 0.001, *** *p* < 0.0001. GraphPad Prism version 10.6.1 was used for data analysis, and the figure panel was created using BioRender: Scientific image and illustration software. Figure S9: To further investigate gene expression overview results in the above genes in the hippocampus (A) Pvalb (C,D) BDNF (E) TrkB, a parametric *t*-test with Welch’s correction was used to confirm significant effects between the two groups. (B) Fgfr2, a non-parametric Mann-Whitney *t*-test was performed as the data was not normally distributed.All data are represented as mean ± SEM and post-hoc significance are presented as: * *p* < 0.01, ** *p* < 0.001, *** *p* < 0.0001.

## Abbreviations

SI	Social isolation
Wt	Wild-type
IgLONs	Immunoglobulin superfamily of cell adhesion molecules
NCAM	Neural cell adhesion molecule
*Negr1*	Neuronal growth regulator 1
*Negr1^−/−^*	Negr1-deficient/knockout
*Gad1*	Glutamic acid decarboxylase 1
*Gad2*	Glutamic acid decarboxylase 2
*Pvalb*	Parvalbumin
*Fgfr2*	Fibroblast growth factor receptor 2
*Bdnf*	Brain-derived neurotrophic factor
*Ntrk2*	Neurotrophic receptor tyrosine kinase 2
*TrkB*	Tropomyosin receptor kinase B
*Ntm 1a/1b*	Neurotrimin 1a/1b
*Lsamp 1a/1b*	Limbic system associated protein 1a/1b
*Cntn1*	Contactin 1
*ActB*	Actin beta
*Gapdh*	Glyceraldehyde-3-phosphate dehydrogenase
*Hprt*	Hypoxanthine-guanine phosphoribosyltransferase 1
*Pgk1*	Phosphoglycerate kinase 1
