# Supplementary material for: Negr1 Deficiency Modulates Sex-Specific Neurobehavioral Adaptations to Social Isolation
Source: Brain Sci. 2025 Nov 29;15(12):1286. doi: 10.3390/brainsci15121286 (PMC12730293; doi:10.3390/brainsci15121286)
Supplement: Supplementary file 1 [file brainsci-15-01286-s001.zip › brainsci-3980896-supplementary.pdf]

Negr1 Deficiency Modulates Sex-Specific Neurobehavioral Adaptations to Social Isolation

Arpana Reinsberg[1], Katyayani Singh[1], Mohan Jayaram[1], Kaie Mikheim[1],

Mari-Anne Philips[1], Eero Vasar[1].

Supplementary figures

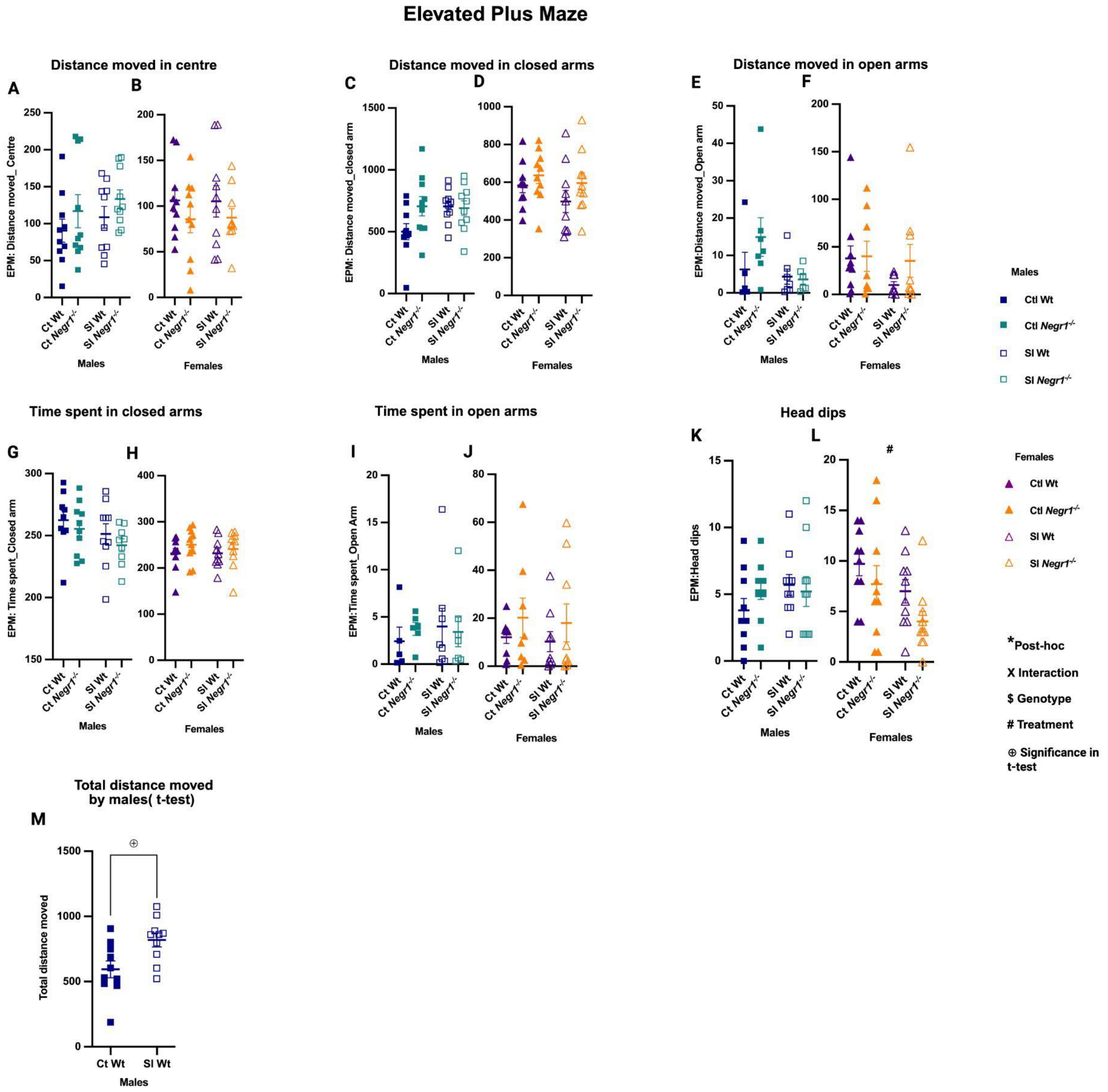

Supplementary figure S1: Activity in Elevated plus maze (EPM) (A-B) Distance moved in centre (C-D) Distance moved in closed arms (E-F) Distance moved in open arms (G-H) Time spent in closed arms

(I-J) Time spent in open arms (K-L) Number of head dips. [Males (n=10), Females (n=10)]. Data were analysed by two-way ANOVA (factors: genotype, treatment) with Bonferroni post hoc tests.
(M) Total distance moved by Ctl and SI Wts, a parametric t-test with Welch's correction was used to confirm significant effects between the two groups. All data are represented as mean ± SEM and post-hoc significance are presented as: ***p<0.01,**p<0.001, ***p<0.0001***.
GraphPad Prism version 10.6.1 was used for data analysis, and the figure panel was created using **BioRender**: Scientific image and illustration software.

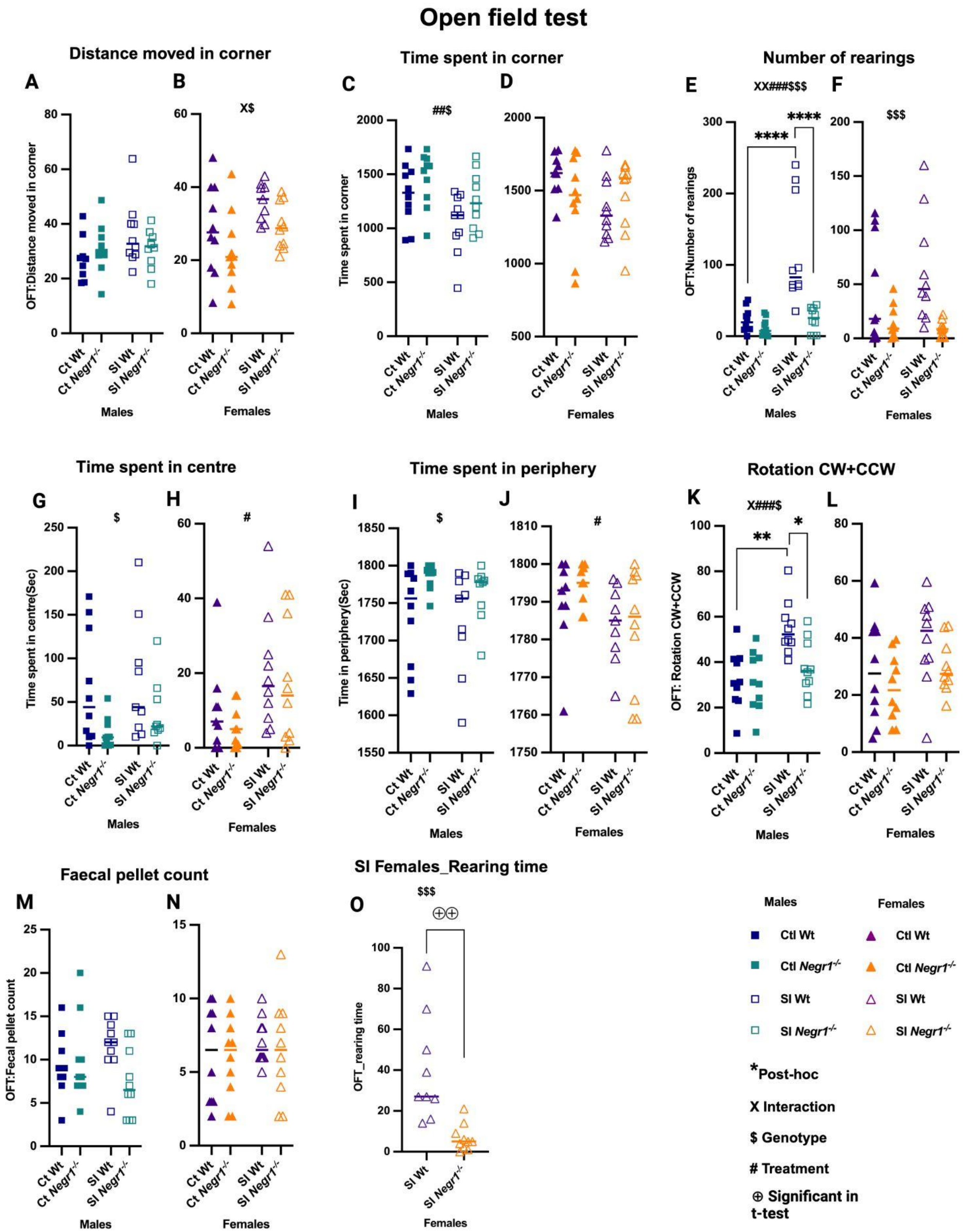
Open field test
Distance moved in corner
A
B
X$
OFT:Distance moved in corner
Time spent in corner
C
D
##$
Time spent in corner
Number of rearings
E
F
XX###$$$
$$$
OFT:Number of rearings
Time spent in centre
G
H
$
#
Time spent in centre(Sec)
Time spent in periphery
I
J
$
#
Time in periphery(Sec)
Rotation CW+CCW
K
L
X###$
OFT: Rotation CW+CCW
Faecal pellet count
M
N
OFT:Fecal pellet count
SI Females_Rearing time
O
$$$
OFT_rearing time
Ct Wt
Ct Negr1-/-
SI Wt
SI Negr1-/-
Males
Females
Ctl Wt
Ctl Negr1-/-
*Post-hoc
X Interaction
$ Genotype
# Treatment
⊕ Significant in t-test

Supplementary figure S2: Activities in Open field test (OFT) (A-B) Distance moved in corner (C-D) Time spent in corner (E-F) Number of rearings (G-H) Time spent in centre (I-J) Time spent in periphery (K-L) Clockwise and counter-clockwise rotations (Rotation CW+CCW)
(M-N) Faecal pellet count during OFT. [Males (n=10), Females (n=10)]. Data were analysed by two-way ANOVA (factors: genotype, treatment) with Bonferroni post hoc tests. (O) Rearing time by SI Wt and SI *Negr1*$^{-/-}$ females, a parametric t-test with Welch's correction was used to confirm significant effects between the two groups. All data are represented as mean ± SEM and post-hoc significance are presented as: **p<0.01,**p<0.001, ***p<0.0001*.
GraphPad Prism version 10.6.1 was used for data analysis, and the figure panel was created using **BioRender**: Scientific image and illustration software.

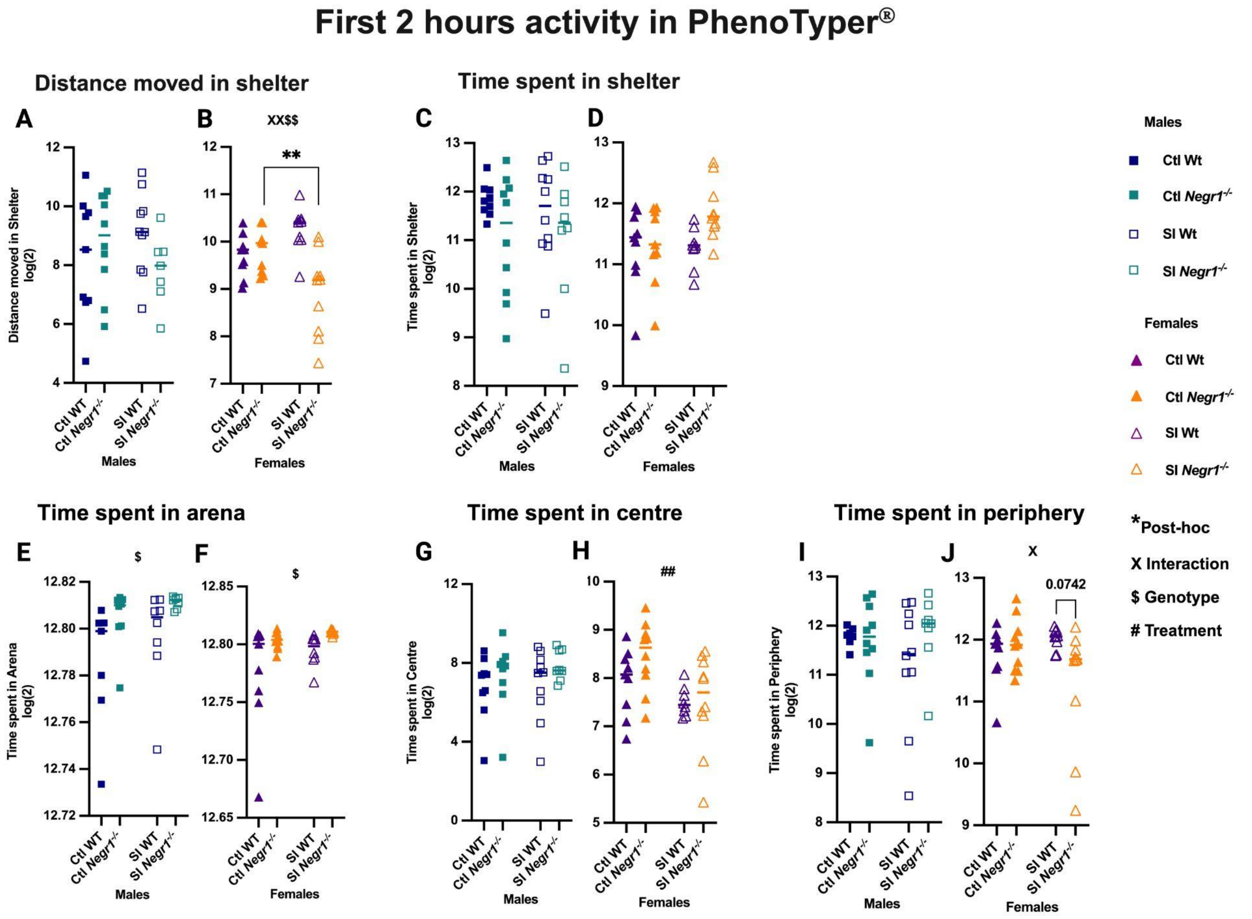

Supplementary figure S3: Activities in shelter and time spent in other arenas. (A-B) Distance moved in shelter (C-D) Time spent in shelter (E-F) Time spent in arena (G-H) Time spent in centre (I-J) Time spent in periphery. [Males (n=10), Females (n=10)]. Data were analysed by two-way ANOVA (factors: genotype, treatment) with Bonferroni post hoc tests. All data are represented as mean ± SEM and post-hoc significance are presented as: ****p<0.01,**p<0.001, ***p<0.0001***.

GraphPad Prism version 10.6.1 was used for data analysis, and the figure panel was created using **BioRender**: Scientific image and illustration software.

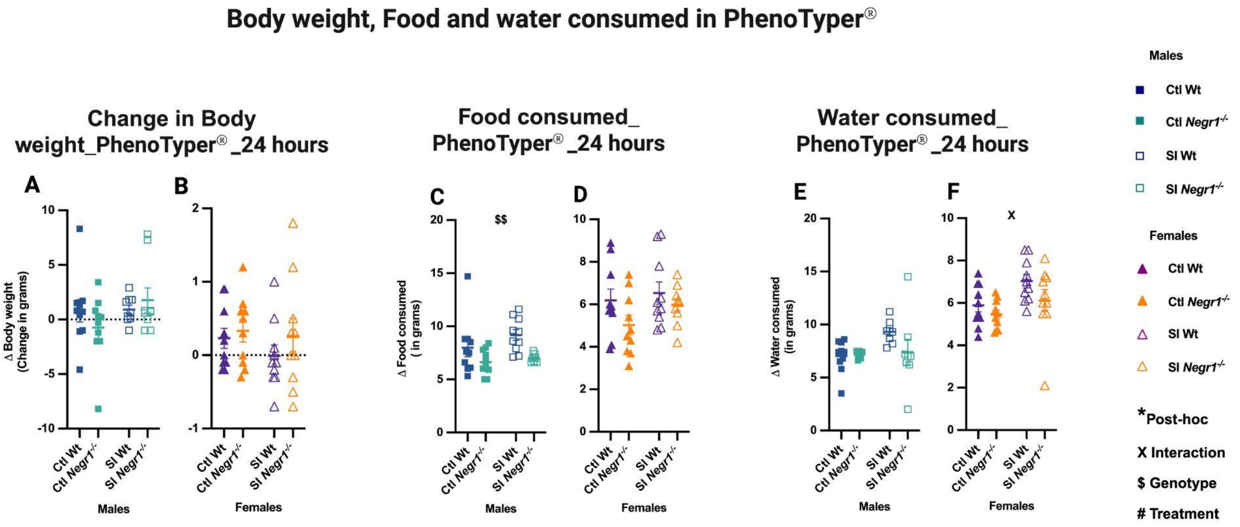

Supplementary figure S4: (A-B) Change in body weight(Δ Body weight) is shown in grams (C-D) Food consumed during 24 hours in PhenoTyper® (E-F) Water consumed during 24 hours in PhenoTyper®. [Males (n=10), Females (n=10)]. Data were analysed by two-way ANOVA (factors: genotype, treatment) with Bonferroni post hoc tests. All data are represented as mean ± SEM and post-hoc significance are presented as: ***p<0.01,**p<0.001, ***p<0.0001***.

GraphPad Prism version 10.6.1 was used for data analysis, and the figure panel was created using **BioRender**: Scientific image and illustration software.

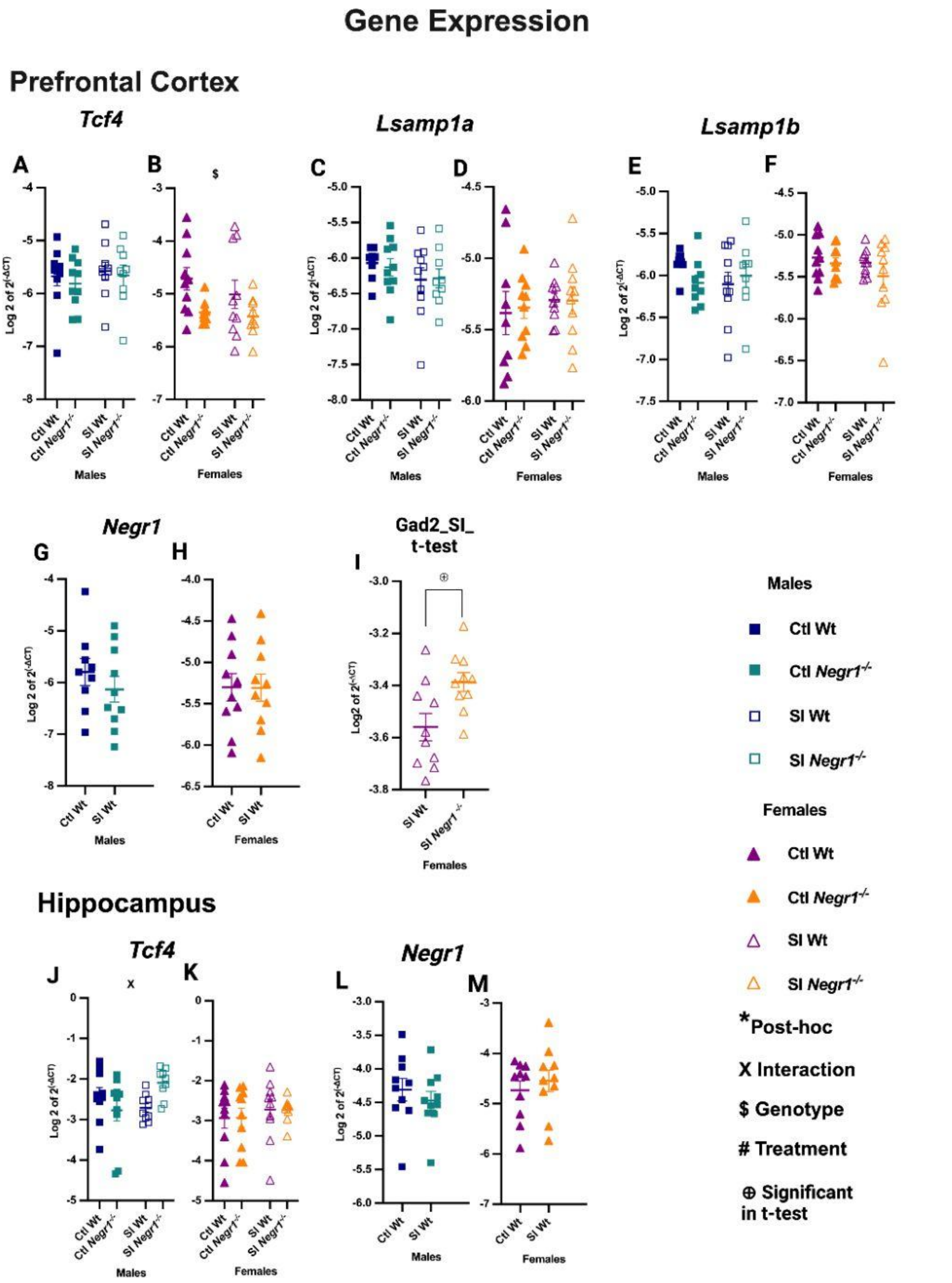
Gene Expression
Prefrontal Cortex
Tcf4
Lsamp1a
Lsamp1b
A
B
$
C
D
E
F
Log 2 of 2^(-ΔCT)
Ctl Wt
Ctl Negr1-/-
SI Wt
SI Negr1-/-
Males
Females
Negr1
G
H
Gad2_SI_
t-test
I
⊕
Log2 of 2^(-ΔCT)
Males
Ctl Wt
Ctl Negr1-/-
SI Wt
SI Negr1-/-
Females
Ctl Wt
Ctl Negr1-/-
SI Wt
SI Negr1-/-
*Post-hoc
X Interaction
$ Genotype
# Treatment
⊕ Significant
in t-test
Hippocampus
Tcf4
Negr1
J
X
K
L
M

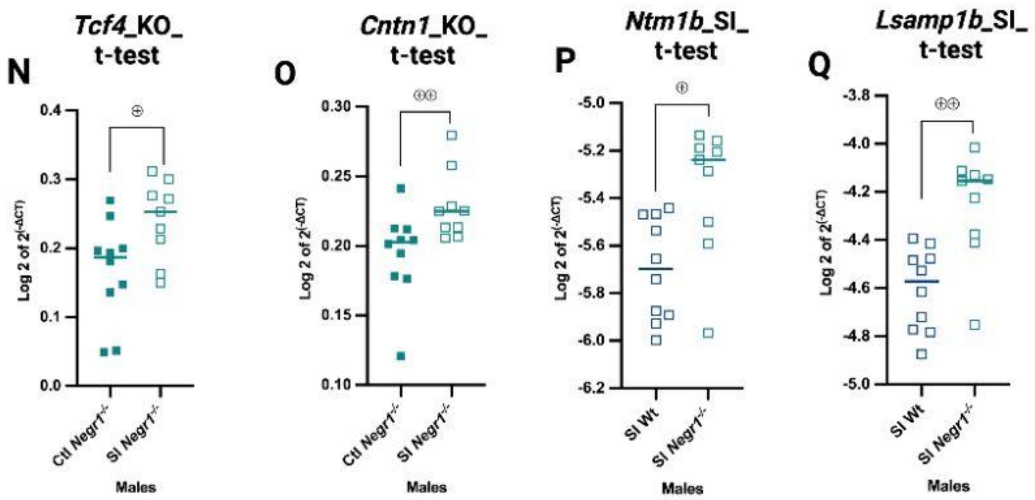

Supplementary figure S5: Gene expression in the prefrontal cortex (A-B) *Tcf4* (C-D) *Lsamp1a* (E-F) *Lsamp1b*. Gene expression in the hippocampus (I-J) *Tcf4* [Males (n=10), Females (n=10)]. Data were analysed by two-way ANOVA (factors: genotype, treatment) with Bonferroni post hoc tests. In the prefrontal cortex (G-H) *Negr1* (I) *Gad2* and in the hippocampus (L-M) *Negr1,* (N) *Tcf4,* (O) *Cntn1,* (P) *Ntm1b,* (Q) *Lsamp1b,* a parametric t-test with Welch's correction was used to confirm significance between two groups when the data were normally distributed, or else a non-parametric Mann-Whitney t-test was performed. All data are represented as mean ± SEM, and post-hoc significance is presented as: ***p<0.01,**p<0.001, ***p<0.0001***.

GraphPad Prism version 10.6.1 was used for data analysis, and the figure panel was created using **BioRender**, a scientific image and illustration software.

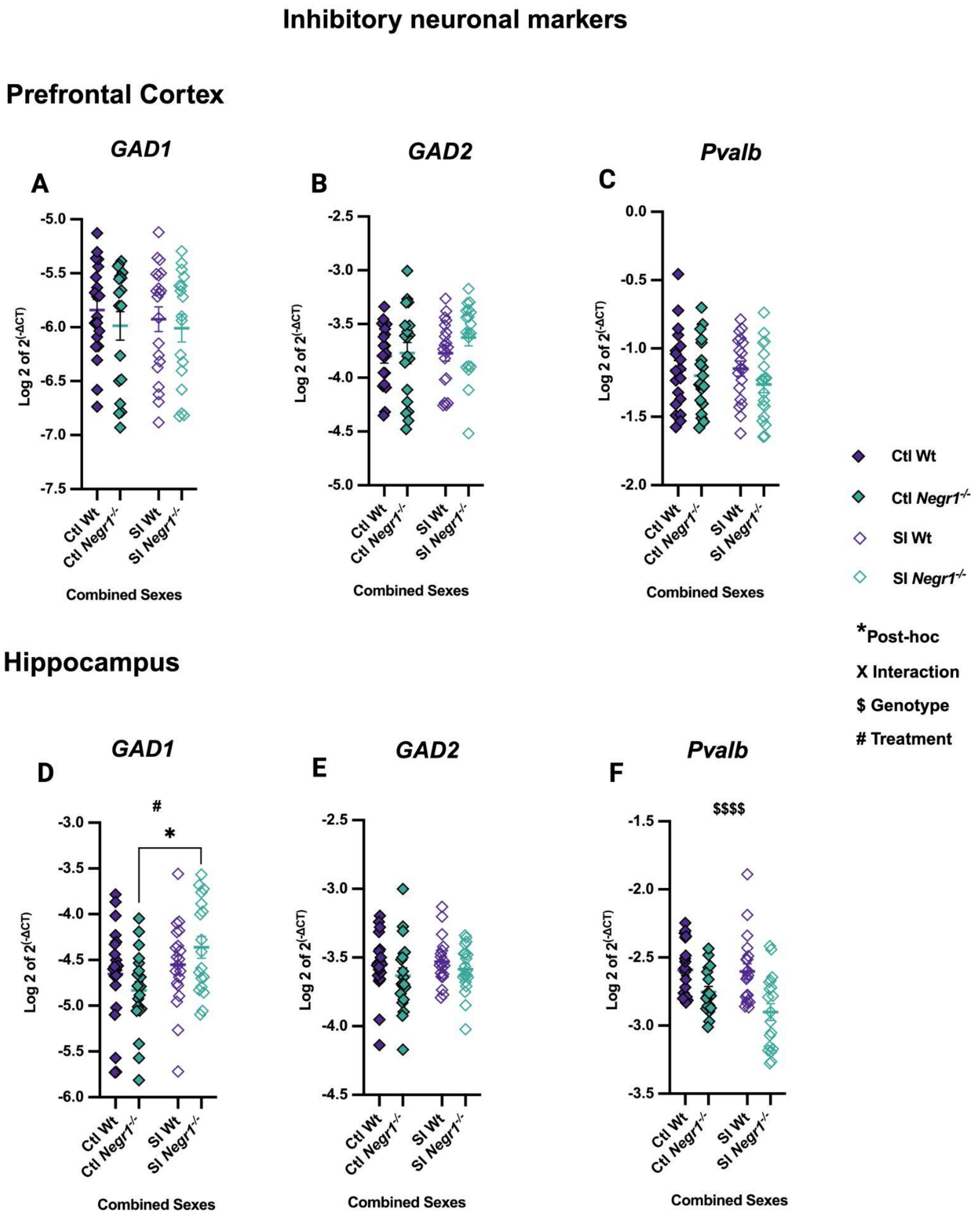

Supplementary figure S6: From the gene expression studies, data from both sexes were combined to get an overview. In the frontal cortex (A) *Gad1* (B) *GAD2* (C) *Pvalb*, in the hippocampus (D) *Gad1* (E) *Gad2* (F) *Pvalb.* [Males (n=10), Females (n=10)]. Data were analysed by two-way ANOVA (factors:

genotype, treatment) with Bonferroni post hoc tests. All data are represented as mean ± SEM and post-hoc significance are presented as: ***p<0.01,**p<0.001, ***p<0.0001***.

GraphPad Prism version 10.6.1 was used for data analysis, and the figure panel was created using **BioRender**: Scientific image and illustration software.

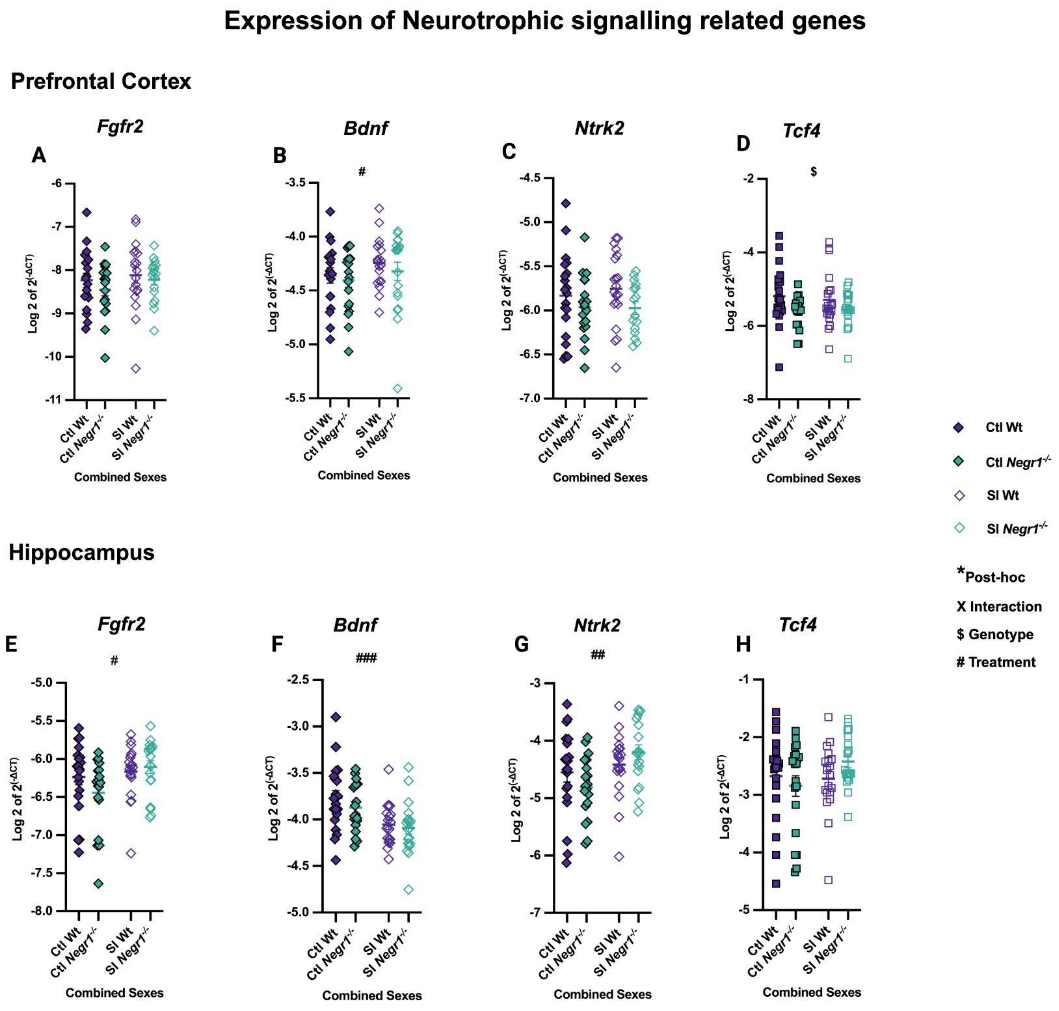

Supplementary figure S7: Expression of neurotropic signalling molecules, data from both sexes were combined to get an overview. In the frontal cortex(A) *Fgfr2* (B) *Bdnf* (C) *Ntrk2* (D) *Tcf4*. In the hippocampus (D) *Fgfr2* (E) *Bdnf* (F) *Ntrk2* (H) *Tcf4* (Males, n=10, females, n=10). Data were analysed by two-way ANOVA (factors: genotype, treatment) with Bonferroni post hoc tests. All data are represented as mean ± SEM and post-hoc significance are presented as: ***p<0.01,**p<0.001, ***p<0.0001***.

GraphPad Prism version 10.6.1 was used for data analysis, and the figure panel was created using **BioRender**: Scientific image and illustration software.

Cell adhesion molecules

Prefrontal Cortex

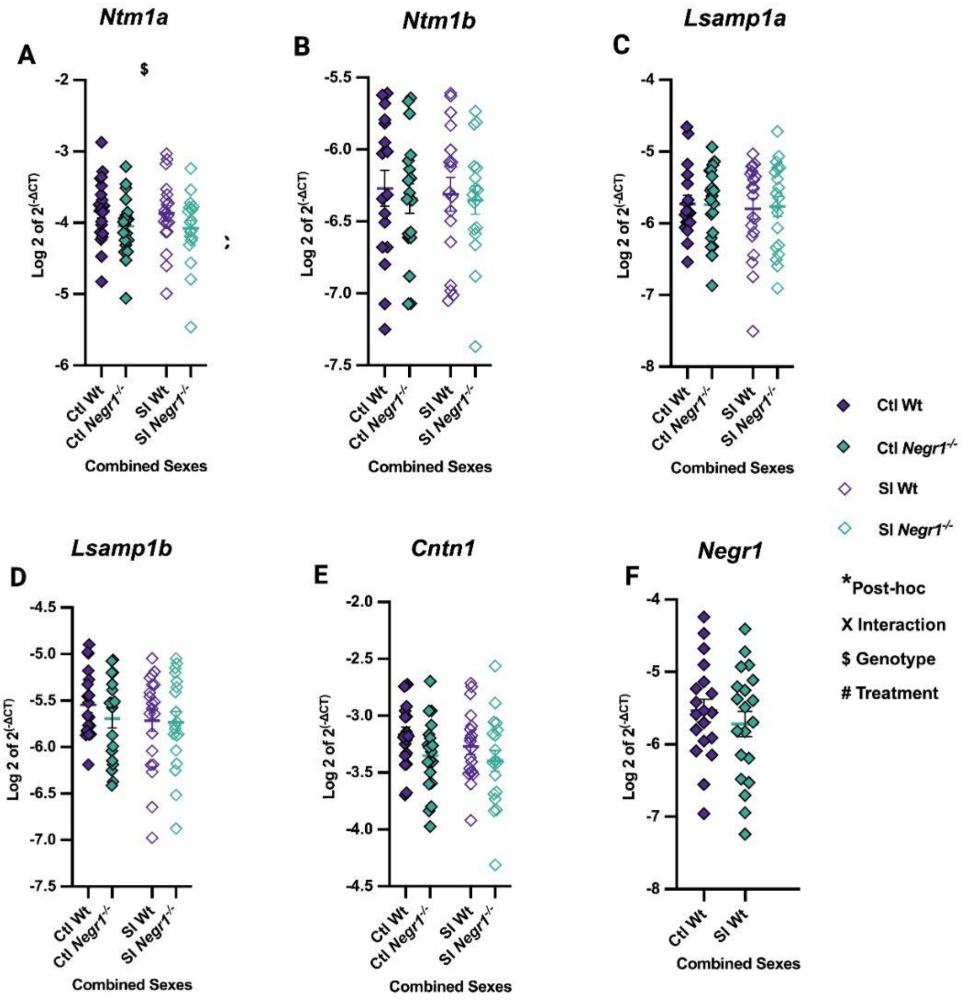

Hippocampus

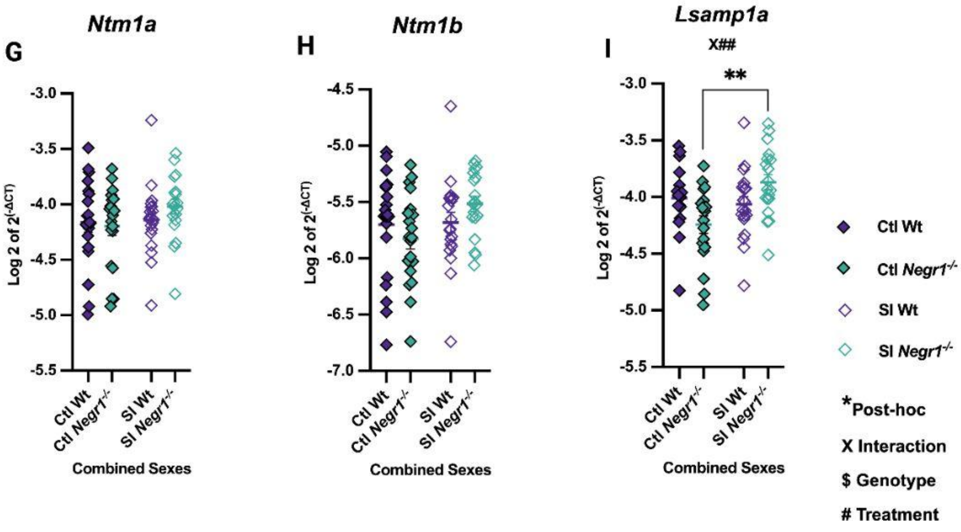

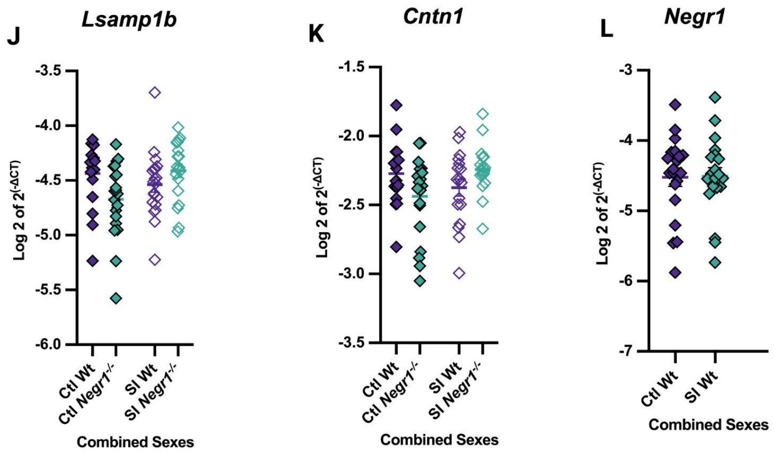

Supplementary figure S8: Expression of IgLONs and *Cntn1* when both sexes are combined. In the frontal cortex (A) *Ntm1a* (B) *Ntm1b* (C) *Lsamp1a* (D) *Lsamp1b* (E) *Cntn1*. In the hippocampus (G) *Ntm1a* (H) *Ntm1b* (I) *Lsamp1a* (J) *Lsamp1b* (K) *Cntn1*. (Males, n=10, females, n=10). Data were analysed by two-way ANOVA (factors: genotype, treatment) with Bonferroni post hoc tests.

(F-L) *Negr1,* a parametric t-test with Welch's correction was used to confirm significant effects between the Wts. All data are represented as mean ± SEM and post-hoc significance are presented as: ***p<0.01,**p<0.001, ***p<0.0001***.

GraphPad Prism version 10.6.1 was used for data analysis, and the figure panel was created using **BioRender**: Scientific image and illustration software.

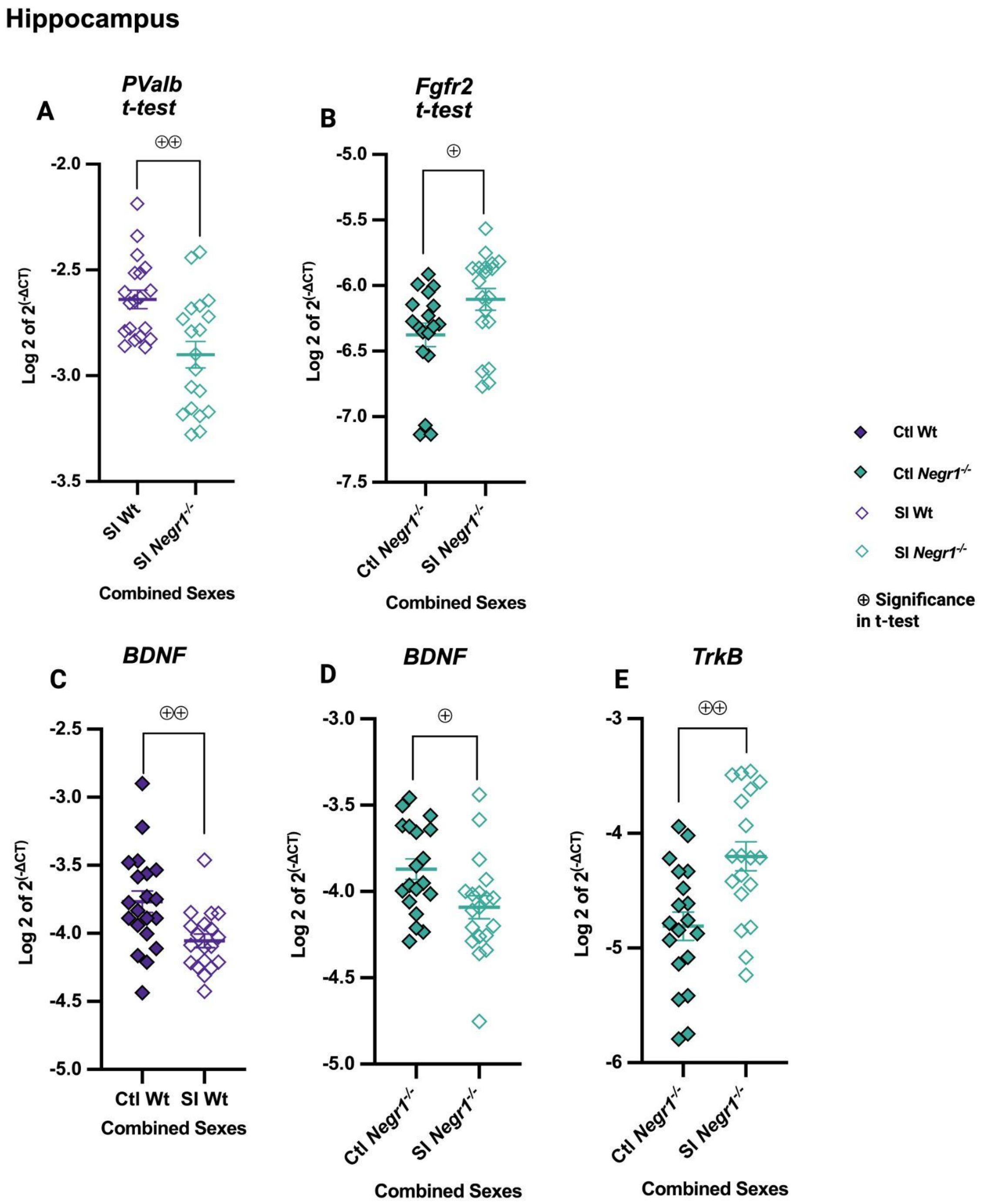

Supplementary figure S9: To further investigate gene expression overview results in the above genes in the hippocampus (A) *Pvalb* (C-D) *BDNF* (E) *TrkB,* a parametric t-test with Welch's correction was used to confirm significant effects between the two groups.

(B) *Fgfr2,* a non-parametric Mann-Whitney t-test was performed as the data was not normally distributed.All data are represented as mean ± SEM and post-hoc significance are presented as: ***p<0.01,**p<0.001, ***p<0.0001***.

GraphPad Prism version 10.6.1 was used for data analysis, and the figure panel was created using **BioRender**: Scientific image and illustration software.
